# Unexpected High Intragenomic Variation in Two of Three Major Pest Thrips Species Does Not Affect Ribosomal Internal Transcribed Spacer 2 (ITS2) Utility for Thrips Identification

**DOI:** 10.3390/ijms18102100

**Published:** 2017-10-06

**Authors:** Vivek Kumar, Aaron M. Dickey, Dakshina R. Seal, Robert G. Shatters, Lance S. Osborne, Cindy L. McKenzie

**Affiliations:** 1 Department of Entomology and Nematology, Mid-Florida Research and Education Center, University of Florida, Apopka, FL 32703, USA; aaron.dickey@ars.usda.gov (A.M.D.); lsosborn@ufl.edu (L.S.O.); 2 U.S. Horticultural Research Laboratory, USDA-ARS, Fort Pierce, FL 34945, USA; Robert.shatters@ars.usda.gov (R.G.S.); cindy.mckenzie@ars.usda.gov (C.L.M.); 3 Department of Entomology and Nematology, Tropical Research and Education Center, University of Florida, Homestead, FL 33031, USA; dseal3@ufl.edu; 4 Present Address: U.S. Meat Animal Research Center, USDA-ARS, Clay Center, NE 68933, USA

**Keywords:** cytochrome oxidase 1 (CO1), internal transcribed spacer (ITS2), intergenomic variation, genetic diversity, chilli thrips, melon thrips, western flower thrips

## Abstract

The mitochondrial cytochrome oxidase I gene (*mtCO1*) and the ribosomal internal transcribed spacer 2 region (ITS2) are among the most widely used molecular markers for insect taxonomic characterization. Three economically important species of thrips, *Scirtothrips dorsalis*, *Thrips palmi*, and *Frankliniella occidentalis* were selected to examine the extent of intragenomic variation within these two marker regions in the family Thripidae, and determine if this variation would affect the utility of markers in thrips molecular diagnostics. For each species, intragenomic (within individual) variation and intergenomic (among individuals) variation was assessed by cloning and sequencing PCR-amplified copies. Intergenomic variation was generally higher than intragenomic variation except in cases where intergenomic variation was very low, as in *mtCO1* from *S. dorsalis* and *F. occidentalis*. Intragenomic variation was detected in both markers in all three of the thrips species, however, 2–3 times more intragenomic variation was observed for ITS2 than *mtCO1* in both *S. dorsalis* and *T. palmi*. Furthermore, levels of intragenomic variation were low for both of the genes in *F. occidentalis*. In all of the three thrips species, no sex-based clustering of haplotypes was observed in either marker. Unexpected high intragenomic variation in ITS2 for two of three thrips species did not interfere with thrips diagnostics. However, caution should be taken in applying ITS2 to certain studies of *S. dorsalis* and *T. palmi* when high levels of intragenomic variation could be problematic or confounding. In such studies, *mtCO1* may be a preferable marker. Possible reasons for discrepancies in intragenomic variation among genomic regions are discussed.

## 1. Introduction

The morphological identification of various species in the order Thysanoptera can be difficult because of their small size (<1.5 mm), high degree of polymorphism within species [1,2,3,4], the similarity of larvae belonging to different species [4,5,6,7,8], and the paucity of thrips taxonomic experts [9]. Furthermore, when cryptic species are present, morphological identification becomes insufficient since an unknown number of species appear morphologically identical [2,10,11]. However, the utility of molecular identification is not affected by the above limitations of morphology based identification of thrips. Molecular identification is economical, fast, and does not require morphological expertise [12]. Various molecular markers have been developed in the past for use in species determination. These include regions of several nuclear genes (18S rRNA encoding and 28S rRNA encoding) [13,14,15] and internal transcribed spacers (rDNA ITSs) [16], as well as the mitochondrial cytochrome c oxidase 1 (*mtCO1*) gene [2].

A portion of the *mtCO1* gene is broadly accepted as an animal DNA barcode for taxon identification, species delimitation, and phylogenetic placement [12]. It is believed to be conserved at the species level and typically displays ≥3% divergence among different species [17,18], making it well suited for this purpose [4]. However, there are cases for several arthropod groups where the use of *mtCO1* for taxon characterization has delivered ambiguous results [19,20,21,22,23,24,25]. Because *mtCO1* and internal transcribed spacer 2 (ITS2) have different modes of evolution and transmission, Navajas et al. [26] used both of the markers to determine whether complimentary or contrasting evolutionary patterns existed among genes. The internal transcribed spacer of the rRNA encoding 5.8S–28S gene cluster is multi-copy due to tandem duplication within the nuclear genome. This marker, like *mtCO1*, is also used for delimiting cryptic species [2] due to low intraspecific variability [27]. This low level of intra-specific variability in the non-coding ITS2 region is assumed to be ensured by concerted evolution [28], where individual members of a multicopy gene cluster do not diverge independently since advantageous mutations are rapidly spread to all members of the cluster [28,29]. In nuclear rDNA, this homogenization of mutations acts as quality control to maintain intragenomic uniformity. Unequal crossing over and gene conversion both drive concerted evolution by repairing mismatches among recombining chromosomes, while gene amplification can select for copies carrying beneficial mutations [28,30]. Nevertheless, several instances of unexpectedly high intragenomic variation in the ITS2 region of arthropods have been reported [26,31,32,33], raising concern about the suitability of this marker for molecular identification. Given that variation may exist in the *mtCO1* and *ITS2* genes of an individual, Polymerase Chain Reaction (PCR) amplifying and sequencing only one-of-many gene copies could lead to misidentification and phylogenetic placement of an individual if intragenomic variation was sufficiently high [18]. Thus, determining the magnitude of intra- and intergenomic variation in the two genes is of paramount importance for any given species.

Worldwide, a large part of the literature dealing with economically important thrips is focused on four major species, i.e., *Frankliniella occidentalis* Pergande (western flower thrips), *Scirtothrips dorsalis* Hood (chilli thrips), *Thrips tabaci* Lindeman (onion thrips), and *Thrips palmi* Karny (melon thrips) [34,35,36,37,38]. These four species are well known for their significant economic impact on agriculture in the United States [39,40,41,42,43,44,45]. They have a wide host-range and cause damage to fruits, leaves and/or flowers of their hosts by feeding. Furthermore, they transmit important and damaging plant viruses. Three of these species, *F. occidentalis*, *S. dorsalis*, and *T. palmi* account for the vectoring of 12 out of 21 species of Tospovirus [40,46], costing growers millions of dollars of damage annually. With the continued global expansion of these thrips vectors, the agriculture sectors in developing countries with limited resources to combat invasive species can suffer disproportionately.

In Thysanoptera, molecular detection techniques are considered a standard identification tool but require a known reference to which unknowns can be compared. The current study was initiated due to unexpected intragenomic variation found while attempting to characterize different populations of *S. dorsalis* with ITS2. One of the goals of the project was to trace the possible origins of invasive Florida *S. dorsalis*, but ITS2 amplification did not reliably produce a single/universal band, and sequencing results were not repeatable. Thus, in the present study we sought to clone and determine the extent of intragenomic variation within this gene and compare it with that found in *mtCO1* for this, and two other globally important thrips species *F. occidentalis* and *T. palmi*. These results are presented with respect to the suitability of the markers in phylogenetic and taxonomic studies. The location of rDNA on thrips is unknown, but whether on autosomes or sex chromosomes [47,48,49,50], males may possess half of the intragenomic ITS2 variation of females because males are haploid. Thus, gender-based differences in ITS2 intragenomic variation in three thrips species was also investigated. Frey and Frey [19] documented a low level of intragenomic variation in the *mtCO1* gene of *T. tabaci* and determined that it would not negatively affect mtDNA-based molecular diagnosis. However, no other published information is available regarding such variation in the two genes of any other thrips species. To our knowledge, this is the first study where intragenomic variation in two genes has been compared across multiple insect genera.

## 2. Results

### 2.1. DNA Sequence Analysis

*Scirtothrips dorsalis*: One hundred thirty-two *mtCO1* clones were sequenced from two females (SD-1, SD-2) and two males (SD-3, SD-4) with 24 to 44 clones/individual (Table 1). The consensus sequence was 655 bp with a GC content of 30.6%. One hundred thirty-two clones produced 21 paralogous haplotypes (Table 1). Forty of 655 sites (6.1%) were variable. Twenty of 21 haplotypes were represented by a single clone, and one haplotype was shared (contained clones from ≥2 individuals). The frequency of the most common haplotype was 84.8% (Table 1). One hundred thirty-seven ITS2 clones were sequenced from four individuals with 23–46 clones/individual. The consensus sequence was 502 bp with a GC content of 55.4%. The 137 clones contained 71 paralogous haplotypes. Eighty-six of 502 (17.1%) sites were variable. Sixty-one of 71 haplotypes were represented by a single clone, and four shared haplotypes were detected. The frequency of the most common haplotype was 10.9% (Table 1).

*Thrips palmi*: One hundred twenty *mtCO1* clones were sequenced from two females (TP-1, TP-2) and two males (TP-3, TP-4) with 24–42 clones/individual (Table 1). The consensus sequence was 655 bp with a GC of 32%. Eleven paralogous haplotypes from 120 clones differed from each other at 20 (3.0%) sites. Nine of 11 haplotypes were represented by a single clone, and one shared haplotype was observed. The frequency of the most common haplotype was 60.8%. One hundred forty-nine ITS2 clones were sequenced from four individuals with 16–28 clones/individual. The consensus sequence was 564 bp with a GC content of 55.1%. One hundred forty-nine clones produced 76 paralogous haplotypes. Haplotypes differed from each other at 79 (14%) sites. Sixty-five of 76 haplotypes were represented by a single clone, and six shared haplotypes were observed. The frequency of the most common haplotype was 14.7% (Table 1).

*Frankliniella occidentalis*: One hundred fifty *mtCO1* clones were sequenced from two females (FO-1, FO-2) and two males (FO-3, FO-4) with 31–46 clones/individual (Table 1). The consensus sequence was 434 bp with a GC content of 34.3%. One hundred fifty clones produced 14 paralogous haplotypes that differed from each other at 22 (5.1%) sites. Nine of 14 haplotypes were represented by a single clone, and one shared haplotype was observed. The frequency of the most common haplotype was 88.6% (Table 1). One hundred five ITS2 clones were sequenced from four individuals with 17–36 clones/individual. The consensus sequence was 454 bp with a GC content of 50.6%. The 105 clones contained 14 paralogous haplotypes. Haplotypes differed from each other at 23 (5.0%) sites. Twelve of 14 haplotypes were represented by a single clone, and one shared haplotype was observed. The frequency of the most common haplotype was 72.3% (Table 1).

**Figure 1:**
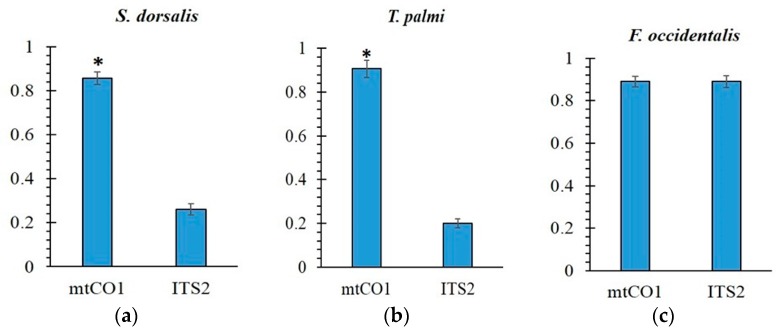
Frequency (*Y*-axis) of the most common sequence (a proxy for the likelihood of obtaining the dominant haplotype in a single pass sequencing effort) for two genes in (**a**) *Scirtothrips dorsalis*, (**b**) *Thrips palmi* and (**c**) *Frankliniella occidentalis*. Given equivalent primer-template binding efficiencies, a researcher is >3× more likely (* *p* = 0.05) to obtain the dominant haplotype of mitochondrial cytochrome c oxidase 1 (*mtCO1*) than internal transcribed spacer 2 (ITS2) in two out of three species.

The likelihood of sequencing the dominant haplotype was significantly higher (>3 times) for *mtCO1* than ITS2 in *S. dorsalis* (*p* = 0.027) and *T. palmi* (*p* = 0.023), whereas no significant differences among genes was observed in *F. occidentalis* (*p* = 1.0) (Figure 1). Sequence variation among different haplotypes of both genes for three species are presented in supplementary.

### 2.2. Intragenomic and Intergenomic Variation

*Scirtothrips dorsalis*: In *S. dorsalis*, the *mtCO1* intragenomic variation ranged between 0.15–0.91% (mean 0.59%) and intergenomic variation ranged between 0.0–0.9% (mean 0.61%) (Table 2). For ITS2, intragenomic variation ranged between 0.20–3.82% (mean 1.45%) and intergenomic variation ranged between 0.0–3.82% (mean 1.83%). Intergenomic variation was significantly higher than intragenomic variation for ITS2 (*p* < 0.002) but not *mtCO1* (*p* = 0.57). Significantly less intragenomic variation was observed for *mtCO1* than for ITS2 (*p* < 0.002). No significant differences in the amount of intragenomic variation among sexes were observed in *mtCO1* or ITS2 (SD-CO1 *p* = 0.4510, SD-ITS *p* = 0.7158).

*Thrips palmi*: In *T. palmi*, the *mtCO1* intragenomic variation ranged between 0.0–0.61% (mean 0.41%) and intergenomic variation ranged between 0.0–1.07% (mean 0.81%) (Table 2). For ITS2, intragenomic variation ranged between 0.18–2.84% (mean 1.26%) and intergenomic variation ranged between 0.0–3.01% (mean 1.37%). Intergenomic variation in *T. palmi* was significantly higher than intragenomic variation for both *mtCO1* (*p* < 0.002) and ITS2 (*p* < 0.002) genes. Significantly less intragenomic variation was observed for *mtCO1* than ITS2 (*p* < 0.002). No significant differences in the amount of intragenomic variation among sexes were observed in *mtCO1* or ITS2 (TP-CO1 *p* = 0.0555, TP-ITS *p* = 0.1297).

*Frankliniella occidentalis.* In *F. occidentalis*, the *mtCO1* intragenomic variation ranged between 0.23–1.15% (mean 0.66%) and intergenomic variation ranged between 0.0–1.38% (mean 0.78%) (Table 2). For ITS2, intragenomic variation ranged between 0.44–1.10% mean (0.74%) and intergenomic variation ranged between 0.0–1.77% (mean 0.98%). Intergenomic variation in this species was significantly higher than intragenomic variation for ITS2 (*p* = 0.004) but not *mtCO1* (*p* = 0.083). Unlike *S. dorsalis* and *T. palmi*, no significant difference in intragenomic variation was observed among genes (*p* = 0.208). No significant differences in the amount of intragenomic variation among sexes were observed in *mtCO1* or ITS2 (FO-CO1 *p* = 0.9293, FO-ITS *p* = 0.7188).

### 2.3. Phylogenetic Analyses

**Figure 2:**
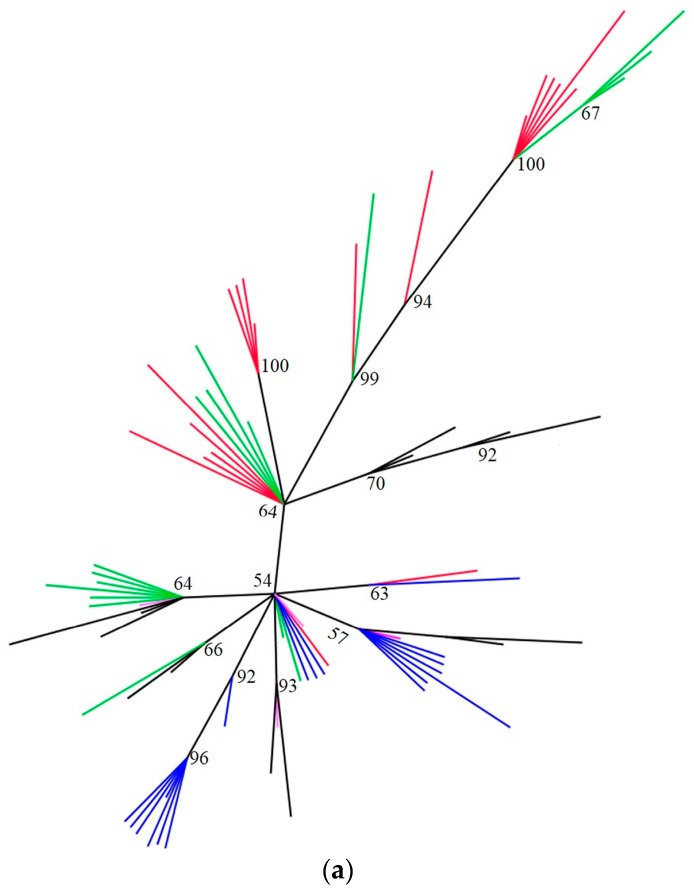

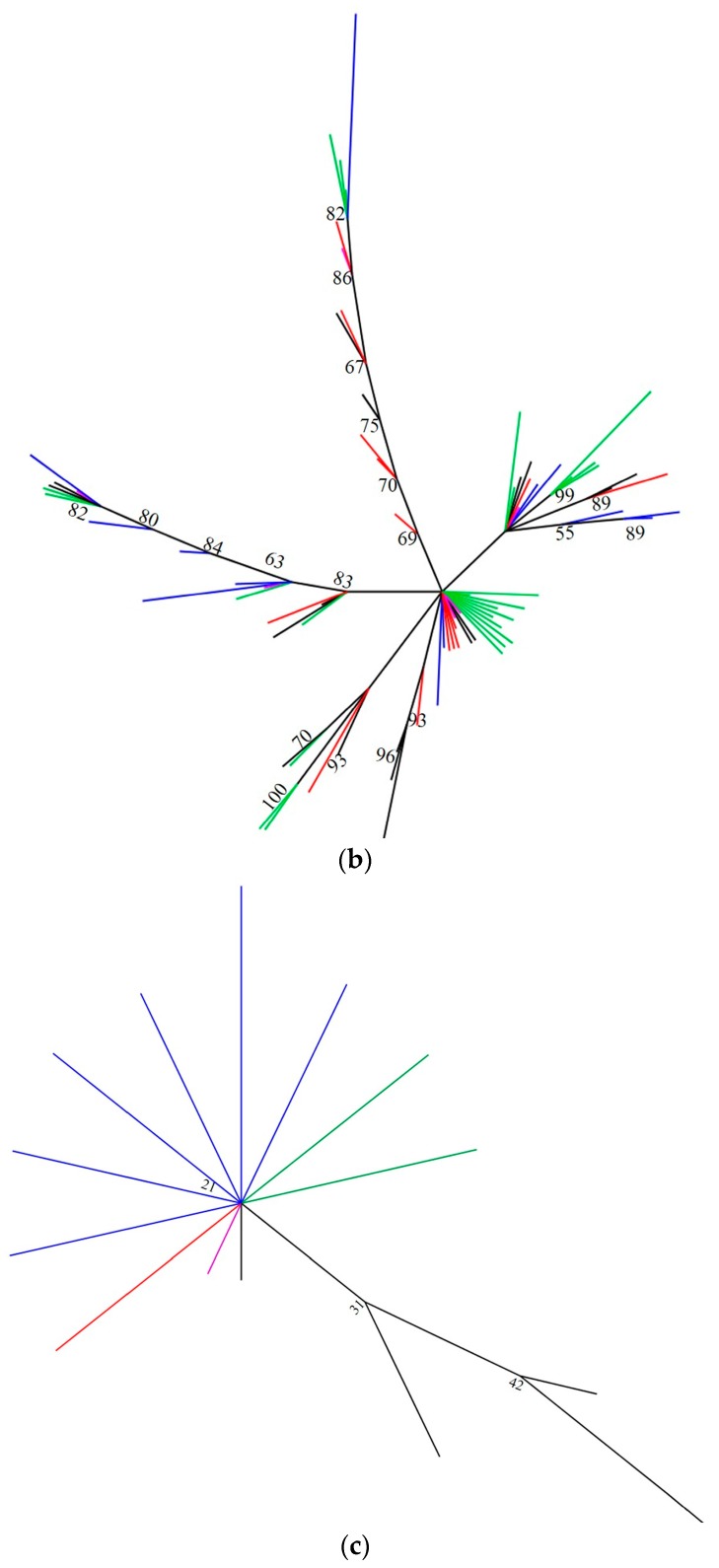
An unrooted Bayesian tree generated from rDNA ITS2 sequence obtained from 2 female and 2 male individuals of (**a**) *Scirtothrips dorsalis*, (**b**) *Thrips palmi* and (**c**) *Frankliniella occidentalis*. Clones from different individuals have been coded in different colors. Bootstrap values are on the branches. Black: Specimen no.1 (SD1, TP1, or FO1), Red: Specimen no. 2 (SD2, TP2, or FO2), Blue: Specimen no. 3 (SD3, TP3, or FO3), Green: Specimen no. 4 (SD4, TP4, or FO4), Pink: Shared haplotype. Number of clones of each specimens included in figures were SD1 = 13, SD2 = 19, SD3 = 18, SD4 = 17 and shared haplotype = 4; TP1 = 17, TP2 = 14, TP3 = 13, TP4 = 26 and shared haplotype = 6; FO1 = 4, FO2 = 1, FO3 = 6, FO4 = 2 and shared haplotype = 1.

The tendency of elevated intragenomic variation in the ITS2 sequence of *S. dorsalis* and *T. palmi* to obscure relationships among individuals can be seen in Figure 2a,b, respectively, where haplotypes do not cluster by individual as would be expected. In contrast, unique clones from an individual cluster together in *F. occidentalis* (Figure 2c).

## 3. Discussion

There is great diversity in the basic biology, life history, host preference, pest status, vector efficiency, and resistance to insecticides in different thrips species, making correct identification critical. Identification provides a link to previously reported biological information about a given species [2] that supports the planning and implementation of scientific research. In the present study, both the marker regions exhibited certain degrees of intragenomic variations, but the variations were not sufficient enough to affect markers’ utility in thrips identification. The significantly lower intragenomic variation in *mtCO1* for two of the three thrips species suggests that this gene is preferable for studies of *S. dorsalis* and *T. palmi*. This is especially true for population genetics studies, where the low likelihood of amplifying the dominant haplotype of ITS2 would be expected to confound accurate parameter estimation. In contrast, both of the markers appear equally well-suited for these types of applications in *F. occidentalis*. The elevated ITS2 intragenomic variation found in *S. dorsalis* and *T. palmi* appears to have two principle consequences, both apparently diminishing utility for molecular characterization: 1) elevated intragenomic variation leads to uncertainty about the “true” or “dominant” sequence of an individual (Figure 1 and Figure 2), elevated intragenomic variation confounds inter-individual relationships (Figure 2a,b), and, as such, may impact population level comparisons within a species. This is consistent with the inference of Srinivasan et al. [51] that ITS2 might not be suitable for such comparisons in a different thrips species, *T. tabaci*, due to the high diversity found within each geographic region.

In the current study, the possibility that *Taq* polymerase induced error could contribute to variation among *mtCO1* and ITS2 clones is valid. Since we used a non-proof-reading *Taq* polymerase lacking the ability of 3′–5′ Īproof reading [52], in standard evaluations of a large number of insects we continued to use the same polymerase. However, we ensured that the variation is real among and within individuals. The *Taq* error rate (mutations per nucleotide per cycle) can vary between 1 × 10^−4^ to 1 × 10^−5^ [33,53,54] and is proportionally related to the length of the product. Thus, considering the number of bases sequenced in the study, artifacts due to *Taq* polymerase account for a small number of clones. In addition, the majority of the unique clones differ by more than a single mutation from other clones (see Supplemental File S1). The possibility of PCR induced error is also low because most mutations are not the result of polymerase slippage (i.e., indel), and most importantly, unexpected high variation was only observed in one of two genes and only in two of the three species under study. This would not be an expected result due to PCR error. The sequence from the same loci of different species are very similar, and therefore variation in *Taq* mutational rates would not be expected among them.

Intragenomic variation in *mtCO1* can be attributed to any of the following factors previously reported by researchers in a variety of arthropods- (i) duplication of the CO1 fragment [55], (ii) nuclear heteroplasmy, where multiple copies of mtDNA undergo coamplification [56], and (iii) nuclear integration of mitochondrial sequences producing pseudogenes (numts) [18]. The first two events are rare phenomena with only a few reports; while, numts (nonfunctional copies of mtDNA) have been found in over 82 species [57]. Among arthropods, they have been reported in aphids, crickets, grasshoppers, and locusts [57,58,59,60,61,62]. These can be co-amplified during PCR resulting in erroneous sequence generation, and consequently, incorrect phylogenetic placement. Our results indicate the co-amplification of numts from total genomic DNA samples using conserved universal primers in the three thrips species. These were identified by the presence of indels, point mutations, or in-frame stop codons. Heteroplasmy was determined as a plausible case for one of the variant *T. palmi* haplotypes from TP-1 that comprised 38 functional clones from the same individual. Similar heteroplasmy was observed in bark weevils, bees, and grasshoppers [18,56,63], and can be inferred by the absence of stop codons in paralogous haplotypes. Nevertheless, we were unable to distinguish between numts and heteroplasmy for most rare haplotypes and the low frequency of variant *mtCO1* haplotypes in the three species did not interfere with molecular diagnostics.

There were a number of base substitutions and indels accounting for sequence variation in ITS2. Based on the significantly elevated intragenomic variation in ITS2 relative to *mtCO1* in *S. dorsalis* and *T. palmi*, Bayesian trees are presented only for this gene (Figure 2a–c). The unrooted Bayesian trees for both species (Figure 2a,b) shows a clustering of clones from the same individual in multiple parts of the tree. Only SD-2 formed its own cluster (Figure 2a), as would be expected. In the case of *T. palmi*, six shared haplotypes were observed and one of the shared haplotypes was found in all four individuals. ITS2 clones from *F. occidentalis* clustered according to individual (Figure 2c), as was the case with all *mtCO1*.

The likely reason behind the variation in ITS2 is gene duplication. Newly duplicated genes can either: (1) evolve independently to produce proteins with new biochemical/physiochemical functions [64] or (2) remain non-functional as pseudogenes in the genome [65]. Importantly, co-amplification of such pseudogenes with the functional copies of ITS2 can bring ambiguity in taxon characterization due to lack of repeatability [66]. In addition, the physical location of duplicated genes on the chromosome can influence its fate, whether for concerted or divergent evolution [28,65]. Even if concerted evolution best explains the data, a faster rate of mutation among copies than the speed of homogenization [27,67] will lead to elevated intragenomic variation.

The high intragenomic variation in ITS2 seen in this study in both *T. palmi* and *S. dorsalis* can be attributed to either a slow rate of concerted evolution or an elevated mutation rate among duplicated genes. Genetic exchange between homologous or non-homologous chromosomes by unequal crossing over or gene conversion [28,30] helps to maintain sequence homogeneity in multigene families, including the rDNA gene family. Mutations generated in one region are either selected against or are rapidly transferred to all of the members of the multigene family, even if repeats are located on different chromosomes [68]. Due to this homogenization of mutations in noncoding regions like ITS2, very low levels of intragenomic and a corresponding fixed rate of intergenomic variation are expected [32]. The existence of 2–3 fold higher intragenomic variation in *S. dorsalis* and *T. palmi* as compared to *F. occidentalis* (Table 2) suggest a higher rate of mutation and/or a lower rate of concerted evolution in these two thrips species. There is indirect evidence of concerted ITS2 evolution in the absence of sex based differences in intragenomic variation. Males, being haploid, should have 1/2 the intragenomic variation of females in the absence of concerted evolution. In a similar study, geographically isolated populations of black fly were reported to exhibit multiple copies of ITS1 [69]. Interbreeding between these populations was interpreted as the source of the multiple ITS1 copies in this pest. Gasser et al. [70] also reported interbreeding populations as the likely source of intragenomic variation in the ITS2 region of the parasitic nematode, *Haemonchus contortus* [31]. When considering the global distribution of *S. dorsalis* and *T. palmi*, interbreeding is a possible cause for the intragenomic variation observed. Future studies should assess whether interbreeding among isolated populations is a plausible explanation of elevated intragenomic variation in ITS2 of *S. dorsalis* and *T. palmi*.

Pseudogenes can also produce variant haplotypes in ITS2, and nonfunctional internal transcribed spacer (ITS) operons or pseudogenes have been documented [71]. In the past, pseudogenes have been reported in *Drosophila melanogaster* Meigen, *Anopheles marajoara* Galvao and Damasceno [32,72], as well as great apes [73] and plants [74]. ITS pseudogenes can be identified by the presence of a high number of indels outside the spacer region as it will affect the structure of ITS2 [75,76]. In this study, a considerable number of indels were found in the haplotypes of *S. dorsalis* suggesting nonfunctional pseudogenes could be playing a role generating intragenomic variation. Future studies on identification of ITS2 pseudogenes in thrips could be aided by an analysis of ITS2 secondary structure [77].

## 4. Materials and Methods

### 4.1. Thrips Sampling

The thrips species used in this study came from populations expected to contain low genetic diversity. *Thrips palmi* specimens originated from an invasive field population that has not spread beyond Orlando, Florida, USA in the 25 years since first reported [42,78]. *Scirtothrips dorsalis* were collected in 2007 from an invasive greenhouse population, two years after it was first documented in Florida, and *F. occidentalis* were sampled from a laboratory colony. Specimen collection permit was not obtained as the thrips species collected on public properties were not endangered or protected. Leaves from thrips infested plants were destructively sampled and placed in a pre-marked Ziplock^®^ (S. C. Johnson & Son, Inc. Racine, WI, USA) bag and transported to the laboratory. Leaves were washed with 75% ethanol to dislodge thrips’ life stages [79]. Thrips adults were carefully removed and placed in 90–95% ethanol and shipped at room temperature to the US Horticultural Research Lab (USHRL-USDA) in Fort Pierce, FL, USA, where samples were stored at −20 °C until analyzed. Information on the host plant, its geographical location, and other collection details are listed in Appendix A (Table A1).

### 4.2. Morphological Identification of Thrips

A single thrips adult was placed in a vial containing 75% ethanol for 10 min, and then transferred for 5 min into another vial with 10% KOH (Potassium hydroxide) solution prepared in 50% ethanol. While in the KOH solution vial, the abdominal region of adults was gently macerated using a fine insect pin to remove abdominal contents. Upon maceration, adults were gradually dehydrated by passing them through a series of ethanol concentration beginning from low to a high concentration in the order of 65%, 75%, 85%, 90%, and 95%, and left for 5–8 min at each concentration. Once dehydrated, each adult was placed ventrally in a drop of Hoyer’s mounting media on a slide and covered with a glass cover slip. The adult specimens were then characterized using morphological features described by Hoddle et al. [80] at a 10× magnification of a dissecting microscope. The specimen’s identity was also confirmed by Thomas Skarlinsky, the thrips taxonomist at USDA-APHIS-Miami, FL.

### 4.3. DNA Processing

Using cohorts from the sample that were morphologically identified, DNA was isolated from two females and two males of each species. Individuals were placed in 1.5-mL Eppendorf tubes with 25 µL of DNA lysis buffer, and ground with a plastic pestle. The pestle was rinsed with an additional 25 µL of DNA lysis buffer and the rinse was collected in the same tube. Tubes were placed in a metal boiling rack and boiled at 95 °C for 5 min and then placed directly in ice for 5 min. The tubes were then centrifuged at 8000× *g* for 30 s and stored at −20 °C until further use. PCR amplifications of the *mtCO1* gene for *S. dorsalis* and *T. palmi* were performed using universal CO1 primers LCO1490 and HCO2198 designed by Folmer et al. [81]. Mitochondrial *CO1* gene amplification for *F. occidentalis* was conducted using mt D-7.2 and mt D-9.2 primers designed by Brunner et al. [5]. Amplification of rDNA ITS2 gene for all of the three species was conducted using Thrips-ITS2 primers [82]. The 25 µL PCR reactions for *CO1* and ITS2 genes consisted of 12.5 µL of Go Taq^®^ Green Mastermix (Promega Corporation, Madison, WI, USA) and 2 µL of DNA template and 10 pmol of each primer. The PCR reactions were run using the conditions described in Appendix A (Table A2), in a PTC-200 Peltier thermal cycler (MJ Research, Watertown, MA, USA). Amplification of the correct PCR products was verified by electrophoresis in a 1.5% agarose gel, stained with ethidium bromide. Before sequencing, the amplified products excised from the gel were cleaned using nucleospin^®^Extract II, PCR clean up, Gel extraction kit (Macherey-Nagel, Inc. Bethlehem, PA, USA). Ligation and transformation of amplified DNA was done using the TOPO^®^ TA Cloning^®^ Kit (Invitrogen, Carlsbad, CA, USA) following the manufacturer’s instructions. Transformed cultures were cultivated in 1.5 mL of Luria-Bertani medium overnight containing 50 ug/mL kanamycin. Plasmids were extracted using the Wizard^®^ Plus SV Minipreps DNA Purification System (Promega Corporation, Madison, WI, USA), dissolved in 0.1× TE and sequenced. All sequencing was performed bi-directionally with the amplification primers and the Prism^®^ BigDye^®^ Terminator v3.1 Cycle Sequencing Kit on a 3730XL DNA Analyzer (both Applied Biosystems, Foster City, CA, USA) at the Genomics Core Instrumentation Facility of USHRL-USDA.

### 4.4. Sequence Alignment and Genetic Variation

Sequence base-calling was verified using Sequencher^™^ 5.0-Build 7081 (Gene Codes Corporation, Ann Arbor, MI, USA) and then aligned using ClustalW 2.1 [83] in Mesquite [84]. Thrips species determination was based on direct sequence comparisons using the web-based National Center for Biotechnology Information (NCBI) BLAST sequence comparison application. In order to minimize possibility of *Taq* random error, single unique mutations (found at a given site in only one sequence) were disregarded. The p-distance among unique clones from the same individual (intragenomic variation) and clones of different individuals of the same species (intergenomic variation) was calculated using DNAsp 5 [85] with gaps treated as a fifth state regardless of size. The frequency of the dominant haplotype of each gene was estimated and used as a proxy for the likelihood of obtaining the dominant haplotype in a single pass sequencing effort as this is the general practice for most studies employing molecular markers diagnostics. Significant differences among the genes and types of genomic variation were tested using Monte-Carlo permutation tests. Intragenomic and Intergenomic variations were also summarized per individual within a species to determine how frequently intragenomic variation exceeds intergenomic variation. Bayesian phylogenetic analysis was carried out using MRBAYES 3.2 [86] to visualize the amount of intragenomic variation across the four individuals representing each gene/species combination. Convergence of the posterior was assessed using the average standard deviation of split frequencies between runs ~0.01, and an effective sample size of each estimated parameter (>200), as determined in Tracer 1.6 [87]. Samples prior to posterior convergence were discarded as burn-in. For each gene/species combination, the analysis was run two times with four chains for 2–3 million generations. Sampling frequency was 1 per 1000 generations and the model was GTR + I + G. The sumtrees script in Dendropy 3.12 [88], Figtree 1.4 [89], and Mesquite program were used for summarizing, viewing, and manipulating trees. Linear mixed models were run to test for differences in intragenomic variation among sexes, with sex as a fixed factor and individual as a random factor in statistical analysis software [90].

## 5. Conclusions

This study quantified intragenomic variation in two genes in three species of thrips representing three pestiferous genera. Although the causes of intragenomic variation in each gene could not be conclusively established in all cases, *mtCO1* numts were found in all of the species and one high frequency *mtCO1* haplotype in *Thrips palmi* may be the result of heteroplasmy. In addition, high indel frequency among ITS2 genevariants suggests pseudogenes as a possible cause of elevated intragenomic variation in *S. dorsalis*. In all three thrips species, no sex-based clustering of haplotypes was observed in ITS2, suggesting similar rDNA arrays in both sexes. We tested whether intragenomic variation in the two markers would undermine molecular diagnostics. Despite unexpectedly high ITS2 variation in two species, all thrips were correctly identified. High levels of intragenomic variation in *S. dorsalis* and *T. palmi* are still potentially problematic because they confounded inter-individual relationships (Figure 2) and dramatically reduce the likelihood of obtaining repeatable results (Figure 1). The ability to obtain repeatable results is critical when purportedly single copy genes are used for certain types of studies, such as population genetics. Given equivalent primer-template binding efficiencies, a researcher is >3× more likely to obtain the dominant haplotype of *mtCO1* than ITS2 in two out of the three species considered. These two species were also representative of two genera, raising the possibility that other thrips genera might show a similar pattern. For *F. occientalis*, both of the markers are robust to the assumption of low intragenomic variation. But for other thrips species such as *S. dorsalis* and *T. palmi*, *mtCO1* may be a preferable marker when the high intragenomic variation is an undesirable marker characteristic. Counter indication of a molecular marker due to intragenomic variation can be exposed by sequencing multiple clones from the same individual and overcome by using alternate markers that better fit the assumptions of a single copy gene.

## Figures and Tables

**Table 1:** Clones sequenced for three thrips species.

	Cytochrome Oxidase 1 (*mtCO1*)			Internal Transcribed Spacer 2 (ITS2)		
Individual ^a^	No. of Clones Sequenced	No. of Different Haplotypes	Freq. of Most Common Haplotype (%)	No. of Clones Sequenced	No. of Different Haplotypes	Freq. of Most Common Haplotype (%)
SD-1	33	4	90.9	23	17	21.7
SD-2	44	11	77.2	26	19	23.0
SD-3	24	4	87.5	42	21	33.3
SD-4	31	5	87.0	46	19	26.0
All *Scirtothrips dorsalis* clones	132	21	84.8	137	71	10.9
TP-1	42	5	90.4	41	23	24.3
TP-2	24	3	91.6	38	18	18.4
TP-3	24	1	100	31	16	22.5
TP-4	30	4	90	39	28	15.3
All *Thrips palmi* clones	120	11	60.8	149	76	14.7
FO-1	42	6	85.7	20	4	85
FO-2	31	4	87.0	17	2	94.1
FO-3	46	5	86.9	36	7	83.3
FO-4	31	2	96.7	32	3	93.7
All *Frankliniella occidentalis* clones	150	14	88.6	105	14	72.3

**^a^** Individual 1 and 2 are female thrips and 3 and 4 are male samples.

**Table 2:** Percentage mean nucleotide divergence (± standard error among unique clones from the same individual (intragenomic variation) and clones of different individuals of the same species (intergenomic variation). Column 4 and 7 is the frequency among individual comparisons where intragenomic variation exceeds a corresponding intergenomic variation.

Thrips Species	Cytochrome Oxidase 1 (*mtCO1*)			Internal Transcribed Spacer 2 (ITS2)		
	Intragenomic Divergence	Intergenomic Divergence	Intragenomic > Intergenomic Var. (%)	Intragenomic Divergence	Intergenomic Divergence	Intragenomic > Intergenomic Var. (%)
*Scirtothrips dorsalis*	0.59 ± 0.02	0.61 ± 0.01	25	1.45 ± 0.03	1.83 ± 0.02	25
*Thrips palmi*	0.41 ± 0.04	0.81 ± 0.04	16.6	1.26 ± 0.02	1.37 ± 0.01	41.6
*Frankliniella occidentalis*	0.66 ± 0.05	0.78 ± 0.04	33.3	0.74 ± 0.05	0.98 ± 0.05	25

## Supplementary Materials

Supplementary materials can be found at www.mdpi.com/1422-0067/18/10/2100/s1.

## Appendix A

**Table A1:** Collection date, localities and hosts for specimens used in cloning of rDNA and *mtCO1* genes of thrips species of three genera.

Scientific Name (M = Male, F = Female)	Specimen No.	Individual Code	Date Collected	Host	Locality	Coordinates	Collector	GenBank Accessions–KT885
*Scirtothrips dorsalis* (F)	CLM9.13	SD-1	7.Aug. 2007	Indian Hawthorne	USA, Florida-Apopka	28.63 N, 81.55 W	Lance Osborne	200, 212
*Scirtothrips dorsalis* (F)	CLM9.14	SD-2	7 Aug. 2007	Indian Hawthorne	USA, Florida-Apopka	28.63 N, 81.55 W	Lance Osborne	201, 213
*Scirtothrips dorsalis* (M)	CLM9.15	SD-3	7 Aug. 2007	Indian Hawthorne	USA, Florida-Apopka	28.63 N, 81.55 W	Lance Osborne	202, 214
*Scirtothrips dorsalis* (M)	CLM9.16	SD-4	7 Aug. 2007	Indian Hawthorne	USA, Florida-Apopka	28.63 N, 81.55 W	Lance Osborne	203, 215
*Thrips palmi* (F)	CLM85.5	TP-1	11 Mar. 2010	Vlaspek cucumber	USA, Florida-Homestead	25.50 N, 80.49 W	Vivek Kumar	204, 216
*Thrips palmi* (F)	CLM85.6	TP-2	11 Mar. 2010	Vlaspek cucumber	USA, Florida-Homestead	25.50 N, 80.49 W	Vivek Kumar	205, 217
*Thrips palmi* (M)	CLM85.9	TP-3	11 Mar. 2010	Vlaspek cucumber	USA, Florida-Homestead	25.50 N, 80.49 W	Vivek Kumar	206, 218
*Thrips palmi* (M)	CLM85.10	TP-4	11 Mar. 2010	Vlaspek cucumber	USA, Florida-Homestead	25.50 N, 80.49 W	Vivek Kumar	207, 219
*Frankliniella occidentalis* (F)	CLM87.20	FO-1	16 Apr. 2011	Green beans	USA, Florida-Tallahassee	30.48 N, 84.17 W	Stuart Reitz	208, 220
*Frankliniella occidentalis* (F)	CLM87.22	FO-2	16 Apr. 2011	Green beans	USA, Florida-Tallahassee	30.48 N, 84.17 W	Stuart Reitz	209, 221
*Frankliniella occidentalis* (M)	CLM87.25	FO-3	16 Apr. 2011	Green beans	USA, Florida-Tallahassee	30.48 N, 84.17 W	Stuart Reitz	210, 222
*Frankliniella occidentalis* (M)	CLM87.30	FO-4	16 Apr. 2011	Green beans	USA, Florida-Tallahassee	30.48 N, 84.17 W	Stuart Reitz	211, 223

**Table A2:** PCR amplification conditions for two genes in *Scirtothrips dorsalis*, *Thrips palmi* and *Frankliniella occidentalis.*

PCR Primer Set	PCR Amplification Conditions (25 µL Reactions)
*mtCO1* primers LCO1490:5′-GGTCAACAAATCATAAAGATATTGG-3′ HCO2198: 5′-TAAACTTCAGGGTGACCAAAAAATCA-3′ * mt D-7.2F: 5′-ATTAGGAGCHCCHGAYATAGCATT-3′ * mt D9.2R: 5′-CAGGCAAGATTAAAATATAAACTTCTG-3′	94 °C 2 min 35 cycles of 94 °C 30 s 54 °C for 30 s 72 °C for 1 min 72 °C for 10 min
ITS2 primers ITSF: 5′-TGTGAACTGCAGGACACATG-3′ ITSR: 5′-AATGCTTAAATTTAGGGGGTA-3′	94 °C 2 min 35 cycles of 94 °C 30 s 52 °C for 1 min 72 °C for 1 min 72 °C for 10 min

* Primers used for *mtCO1* amplification of *Frankliniella occidentalis* with annealing temperature of 52 °C. Annealing temperature for *CO1* and *ITS2* amplification of *Thrips palmi* was 40 and 48 °C, respectively.
