# Supplementary material for: Unexpected High Intragenomic Variation in Two of Three Major Pest Thrips Species Does Not Affect Ribosomal Internal Transcribed Spacer 2 (ITS2) Utility for Thrips Identification
Source: Int J Mol Sci. 2017 Oct 6;18(10):2100. doi: 10.3390/ijms18102100 (PMC5666782; doi:10.3390/ijms18102100)
Supplement: Supplementary file 1 [file ijms-18-02100-s001.pdf]

## *Scirtothrips dorsalis* - ITS

No. of clone/Total clone — Nucleotide Position

| Specimen | Ratio | 1 | 6 | 12 | 32 | 43 | 79 | 84 | 99 | 100 | 101 | 107 | 109 | 111 | 112 | 113 | 126 | 133 | 141 | 142 | 143 | 144 | 147 | 181 | 184 | 190 | 205 | 206 | 219 | 222 | 224 | 233 | 234 | 236 | 237 | 241 | 242 | 250 | 253 | 258 | 262 | 268 | 275 | 282 | 293 | 296 | 302 | 303 | 318 | 324 | 335 | 338 | 344 | 348 | 355 | 365 | 371 | 377 | 380 | 384 | 386 | 393 | 394 | 398 | 401 | 403 | 408 | 412 | 414 | 416 | 417 | 423 | 430 | 431 | 436 | 437 | 439 | 447 | 451 | 452 | 459 | 460 | 461 | 462 | 473 | 477 | 485 | 495 |
|---|---|---|---|---|---|---|---|---|---|---|---|---|---|---|---|---|---|---|---|---|---|---|---|---|---|---|---|---|---|---|---|---|---|---|---|---|---|---|---|---|---|---|---|---|---|---|---|---|---|---|---|---|---|---|---|---|---|---|---|---|---|---|---|---|---|---|---|---|---|---|---|---|---|---|---|---|---|---|---|---|---|---|---|---|---|---|---|---|
| SD-1.1 | 1/137 |A|C|T|C|T|A|A|G|C|G|C|A|A|-|C|T|T|T|T|T|G|C|G|T|T|C|G|C|T|G|T|T|A|C|G|T|C|A|T|G|T|G|A|G|C|C|G|C|A|C|A|C|T|C|C|A|A|C|C|G|C|G|A|A|T|A|T|T|-|-|A|T|T|T|G|G|C|G|C|T|C|C|C|G|T|T|T|
| SD-1.2 | 1/137 |A|T|T|C|T|A|A|G|C|G|T|A|A|A|C|T|T|T|-|C|-|C|G|C|T|C|G|C|T|G|T|T|A|C|G|T|C|A|G|G|T|G|A|G|C|C|G|C|A|C|A|T|T|C|C|A|A|C|C|G|C|G|A|A|T|A|T|T|-|-|A|T|T|T|G|G|C|G|C|T|C|C|C|G|T|T|T|
| SD-1.3 | 1/137 |A|T|T|C|T|A|A|G|C|G|C|A|A|-|C|T|T|T|T|T|G|C|G|T|T|C|G|C|T|G|T|T|A|C|G|T|C|A|G|G|T|G|A|G|C|C|G|C|A|C|A|T|T|C|C|A|A|C|C|G|C|G|A|A|T|A|T|T|-|-|A|T|T|T|G|G|C|G|C|T|C|C|C|G|T|T|T|
| SD-1.4 | 1/137 |A|C|T|C|T|A|A|G|C|G|T|A|A|A|C|T|T|T|-|C|-|C|G|C|T|C|G|C|T|G|T|T|A|C|G|T|C|G|G|G|T|G|A|G|C|C|G|C|A|C|A|T|T|C|C|A|A|C|C|A|C|G|A|A|T|A|T|T|-|-|A|T|T|T|G|G|C|G|C|T|C|C|C|G|T|T|T|
| SD-1.5 | 1/137 |A|C|T|C|T|A|A|-|-|-|T|C|A|-|-|T|T|T|-|C|-|C|G|T|T|C|G|C|T|G|T|T|A|C|G|T|C|A|G|G|T|G|A|G|C|C|G|C|A|C|G|T|T|C|C|A|A|C|C|G|C|G|A|A|T|A|T|T|-|-|A|T|T|T|G|G|C|G|C|T|C|T|C|G|T|T|T|
| SD-1.6 | 1/137 |A|C|T|C|T|A|A|-|-|-|T|C|A|-|-|T|T|T|-|C|-|C|G|T|T|C|G|C|T|G|T|T|A|C|G|T|C|A|G|G|T|G|A|G|C|C|G|C|A|C|G|T|T|C|C|A|A|C|C|G|C|G|A|A|T|A|T|T|-|-|A|T|A|T|G|G|C|G|C|T|C|C|C|G|T|T|T|
| SD-1.7 | 1/137 |A|C|T|C|T|A|A|-|-|-|T|C|A|-|-|T|T|T|-|C|-|C|G|T|T|C|G|C|T|G|T|T|A|C|G|T|C|A|G|G|T|G|A|G|C|C|G|C|A|C|G|T|T|C|C|A|A|C|C|G|C|G|A|A|T|A|T|T|-|-|A|T|T|T|G|G|C|G|C|T|C|C|C|G|T|T|T|
| SD-1.8 | 2/137 |A|C|T|C|T|A|A|G|C|G|C|A|A|-|C|T|T|T|T|T|G|C|G|T|T|C|G|C|T|G|T|T|A|C|G|T|C|A|G|G|T|G|A|G|C|C|G|C|A|C|A|T|T|C|C|A|A|C|C|G|C|G|A|A|T|A|T|T|-|-|A|T|T|T|G|G|C|G|C|T|C|C|C|G|T|T|T|
| SD-1.9 | 1/137 |A|C|T|C|T|A|A|G|C|G|-|A|A|A|C|T|T|T|-|C|-|C|G|T|T|C|G|C|T|G|T|T|A|C|G|T|C|A|G|G|T|G|A|G|C|C|G|C|A|C|G|T|T|C|C|G|A|C|C|G|C|G|A|A|T|A|T|T|-|-|A|T|A|T|G|G|C|G|C|T|C|C|C|G|T|T|T|
| SD-1.10 | 1/137 |A|C|T|C|T|A|A|G|C|G|-|A|A|A|C|T|T|T|-|C|-|C|G|T|T|C|G|C|T|G|T|T|A|C|G|T|C|A|G|G|T|G|A|G|C|C|G|C|A|C|A|T|T|C|C|A|A|C|C|G|C|G|A|A|T|A|T|T|-|-|A|T|T|T|G|G|C|G|C|T|C|T|C|G|C|T|T|
| SD-1.11 | 1/137 |A|C|T|C|T|A|A|G|C|G|T|A|A|A|C|T|T|T|-|C|-|C|G|T|T|C|G|C|T|G|T|T|A|C|G|T|C|A|G|G|T|G|A|G|C|C|G|C|A|C|A|T|T|C|C|A|A|C|C|G|C|G|A|A|T|A|T|T|-|-|A|T|T|T|G|G|C|G|C|T|C|T|C|G|T|T|T|
| SD-1.12 | 1/137 |A|C|T|C|T|A|A|G|C|G|C|A|A|-|C|T|T|T|T|C|G|C|G|T|T|C|G|C|T|G|T|T|A|C|G|T|C|A|T|G|T|G|A|G|C|C|G|C|A|C|A|T|T|C|C|A|A|C|C|G|C|G|A|A|T|A|T|T|-|-|A|T|T|T|G|G|C|G|C|T|C|C|C|G|T|T|T|
| SD-1.13 | 1/137 |A|C|T|C|T|A|A|G|C|G|-|A|A|A|C|T|T|T|-|C|-|C|G|T|T|C|A|C|T|G|T|T|A|C|G|T|C|A|G|G|T|A|A|G|C|C|G|C|A|C|A|T|T|C|C|A|G|C|C|G|C|G|A|A|T|A|T|T|-|-|A|T|T|T|G|G|C|G|C|T|C|C|C|G|T|T|T|
| SD-2.1 | 1/137 |A|C|T|C|T|A|A|-|-|-|T|C|A|-|-|T|T|T|-|C|-|C|G|T|T|C|G|C|T|G|T|T|A|C|G|T|C|A|G|G|T|G|A|G|C|C|G|C|A|C|A|T|T|C|C|A|A|T|C|G|C|G|A|A|T|A|A|C|C|T|A|T|T|T|G|G|C|G|C|T|C|C|C|G|T|T|T|
| SD-2.2 | 6/137 |A|C|T|C|T|A|A|G|C|G|T|A|A|A|C|T|T|T|-|C|-|C|G|T|T|C|G|C|T|G|T|T|A|C|G|T|C|A|G|G|T|G|A|G|C|C|G|C|A|T|A|T|C|C|C|A|A|C|C|G|C|G|A|A|T|A|A|C|C|T|A|T|T|T|G|G|C|G|C|T|C|C|A|G|T|T|C|
| SD-2.3 | 1/137 |A|C|T|C|T|A|A|-|-|-|T|C|A|-|-|T|C|T|T|C|G|C|G|T|T|C|G|C|T|G|T|T|A|T|G|T|C|A|G|G|T|G|A|G|C|C|G|C|A|C|A|T|T|C|C|A|A|C|C|G|C|G|A|A|T|A|T|T|-|-|A|T|T|C|G|G|A|G|C|T|C|C|C|G|T|T|T|
| SD-2.4 | 1/137 |A|C|T|C|T|A|A|-|-|-|T|C|A|-|-|T|T|T|-|C|-|C|G|T|T|C|G|C|T|G|T|T|A|C|G|T|C|A|G|G|T|G|G|G|C|C|G|C|A|C|A|T|T|C|C|A|A|T|C|G|C|G|A|A|T|A|A|C|C|T|A|T|T|T|G|G|C|G|C|T|C|C|C|G|T|T|T|
| SD-2.5 | 2/137 |A|C|T|C|T|A|A|-|-|-|T|C|A|-|-|T|T|T|-|C|-|C|G|T|T|C|G|C|T|G|T|T|T|C|G|T|C|A|G|G|T|G|A|G|C|C|G|C|A|C|A|T|T|C|C|A|A|C|C|G|C|G|A|A|T|A|T|T|-|-|-|T|T|T|G|G|C|G|C|T|C|C|C|G|T|T|T|
| SD-2.6 | 1/137 |A|C|T|C|T|A|A|G|C|G|T|A|A|A|C|T|T|T|-|C|-|C|G|T|T|C|G|C|T|G|T|T|A|C|A|T|C|A|G|G|T|G|A|G|C|C|G|C|A|T|A|T|C|C|C|A|A|C|C|G|C|G|A|A|T|A|A|C|C|T|A|T|T|T|G|G|C|G|C|T|C|C|A|G|T|T|C|
| SD-2.7 | 1/137 |A|C|T|C|T|A|A|-|-|-|T|C|A|-|-|T|T|T|-|C|-|C|G|T|T|C|G|C|T|G|T|T|A|C|G|T|C|A|G|G|T|G|A|G|C|C|G|C|A|C|A|T|T|A|C|A|A|C|C|G|C|G|A|A|T|A|T|T|-|-|A|T|T|T|G|G|C|G|C|T|C|C|G|G|T|T|C|
| SD-2.8 | 2/137 |G|C|T|C|T|A|A|G|C|G|T|A|A|A|C|T|T|T|-|C|-|C|G|T|T|C|G|C|T|G|T|T|A|C|G|T|C|A|G|G|T|G|A|G|C|C|G|C|A|T|A|T|C|C|C|A|A|C|C|G|C|G|A|A|T|A|A|C|C|T|A|T|T|T|G|G|C|G|C|T|C|C|A|G|T|T|C|
| SD-2.9 | 1/137 |A|C|T|C|T|A|A|-|-|-|T|C|A|-|-|T|T|T|-|C|-|C|G|T|T|C|G|C|T|G|T|T|A|C|G|T|C|A|G|G|T|G|A|G|C|C|G|C|A|C|A|T|T|C|C|A|A|T|C|G|C|A|A|A|T|A|A|C|C|T|A|T|T|T|G|G|C|G|C|T|C|C|C|G|T|T|T|
| SD-2.10 | 1/137 |A|C|T|C|T|A|A|G|C|G|T|A|A|A|C|T|T|T|-|C|-|C|G|T|T|T|G|C|T|G|T|T|A|C|G|T|C|A|G|G|T|G|A|G|C|C|G|C|A|T|A|T|C|C|C|A|A|C|C|G|C|G|A|A|T|A|A|C|C|T|A|T|T|T|G|G|C|G|C|T|C|C|A|G|T|T|C|
| SD-2.11 | 1/137 |A|C|T|C|T|A|A|-|-|-|T|C|A|-|-|T|T|T|-|C|-|C|G|T|T|C|G|C|T|G|T|T|A|C|G|T|C|A|G|G|T|G|A|G|C|T|G|C|A|C|A|T|T|C|C|A|A|T|C|G|C|G|A|A|T|A|A|C|C|T|A|T|T|T|G|G|C|G|C|T|C|C|C|G|T|T|T|
| SD-2.12 | 1/137 |A|C|T|C|T|A|A|-|-|-|T|C|A|-|-|T|T|T|-|C|-|C|G|T|T|C|G|C|T|T|T|T|A|C|G|T|C|A|G|G|T|G|A|G|C|C|G|C|A|C|A|T|T|C|C|A|A|C|C|G|C|G|A|A|T|A|T|T|-|-|A|T|T|T|G|G|C|G|C|T|C|C|C|G|T|T|T|
| SD-2.13 | 1/137 |A|C|T|C|T|A|A|G|C|G|T|A|A|A|C|T|T|T|-|C|-|C|G|T|T|C|G|C|T|G|T|T|A|C|G|T|C|A|G|G|T|G|A|G|C|C|G|C|A|T|A|T|C|C|C|A|A|C|C|G|C|G|G|G|T|A|A|C|C|T|A|T|T|T|G|G|C|G|C|T|C|C|A|G|T|C|C|
| SD-2.14 | 1/137 |A|C|T|C|T|A|A|-|-|-|T|C|A|-|-|T|T|T|-|C|-|C|G|T|T|C|G|C|T|G|T|T|A|C|G|T|T|A|G|G|T|G|A|G|T|C|G|C|A|C|A|T|T|C|C|A|A|C|C|G|C|G|A|A|T|A|T|T|-|-|A|T|T|T|G|G|C|G|C|T|C|C|C|G|T|T|T|
| SD-2.15 | 1/137 |A|C|T|C|T|A|A|G|C|G|-|A|A|A|C|T|T|T|-|C|-|C|G|T|T|C|G|C|T|G|T|T|A|C|G|T|C|A|G|G|T|G|A|G|C|C|G|C|A|C|A|T|T|C|C|A|A|C|C|G|C|G|A|A|T|A|T|T|-|-|A|T|T|T|G|G|C|G|C|T|A|C|C|G|T|T|T|
| SD-2.16 | 1/137 |A|C|T|C|T|A|A|-|-|-|T|C|A|-|-|T|T|T|-|C|-|C|G|T|T|C|G|C|T|G|T|T|A|C|G|T|C|A|G|G|T|G|A|G|T|C|G|C|A|C|A|T|T|C|C|A|A|C|C|G|C|G|A|A|T|A|A|C|C|T|A|T|T|T|G|G|C|G|C|T|C|C|A|A|T|T|C|
| SD-2.17 | 1/137 |A|C|T|C|T|A|A|G|C|G|T|A|A|-|C|T|T|T|T|C|G|C|G|T|T|C|G|C|T|G|T|T|A|C|G|T|C|A|G|G|T|G|A|G|C|C|G|C|A|C|A|T|T|C|C|A|A|C|C|G|C|G|A|A|T|A|T|T|-|-|A|A|T|T|G|G|C|G|C|T|C|C|C|G|T|T|T|
| SD-2.18 | 1/137 |A|C|T|T|T|A|A|-|-|-|T|C|A|-|-|T|C|C|-|C|-|C|G|T|T|C|G|C|T|G|T|T|A|C|G|T|C|A|G|G|T|G|A|G|T|C|G|C|A|C|A|T|T|C|C|A|A|C|C|G|C|G|A|A|T|A|T|T|-|-|A|T|T|T|G|G|C|G|C|T|C|C|C|G|T|T|T|
| SD-2.19 | 1/137 |A|C|T|C|T|A|A|G|C|G|T|A|A|A|C|T|T|T|-|C|-|C|G|T|T|C|G|C|T|G|C|T|A|C|G|T|C|A|G|G|T|G|A|G|C|C|G|C|A|T|A|T|C|C|C|A|A|C|C|G|C|G|A|A|T|A|A|C|C|T|A|T|T|T|G|G|C|G|C|T|C|C|A|G|T|T|C|
| SD-3.2 | 14/137 |A|C|T|C|T|A|A|G|T|G|-|A|A|A|C|T|T|T|-|C|-|C|G|T|T|C|G|C|T|G|T|T|A|C|G|T|C|A|G|G|T|G|A|G|C|C|G|C|A|C|T|T|T|C|C|A|A|C|C|G|C|G|A|A|T|A|T|T|-|-|A|T|T|T|G|G|C|G|C|T|C|C|C|G|T|T|T|
| SD-3.3 | 1/137 |A|C|T|C|T|A|A|G|C|G|C|A|A|-|C|T|T|T|T|C|A|C|G|T|T|C|G|C|T|G|T|T|A|C|G|T|C|A|G|G|T|G|A|G|C|C|G|C|A|C|T|T|T|C|C|A|A|C|C|G|C|G|A|A|T|A|T|T|-|-|A|T|T|T|G|G|C|G|C|T|C|C|C|G|T|T|C|
| SD-3.5 | 1/137 |A|C|T|C|T|A|A|G|C|G|-|A|A|A|C|T|T|T|T|C|G|C|G|T|T|C|G|C|T|G|T|T|A|C|G|T|C|A|T|G|T|G|A|G|C|C|G|C|A|C|A|T|T|C|C|A|A|C|C|G|C|G|A|A|C|A|T|T|-|-|A|T|T|T|G|G|C|G|C|T|C|C|C|G|T|T|T|
| SD-3.6 | 1/137 |G|C|T|C|T|A|A|G|C|G|-|A|A|A|C|T|T|T|T|C|G|C|G|T|T|C|G|C|T|G|T|T|A|C|G|T|C|A|T|G|T|G|A|G|C|C|G|C|A|C|A|T|T|C|C|A|A|C|C|G|C|G|A|A|T|A|T|T|-|-|A|T|T|T|G|G|C|G|C|T|C|C|C|G|T|T|T|
| SD-3.7 | 1/137 |A|C|T|C|T|A|A|G|T|G|-|A|A|A|C|T|T|T|-|C|-|C|G|T|T|C|G|C|T|G|T|T|A|C|G|T|C|A|G|G|T|G|A|G|C|C|G|C|A|C|T|T|T|C|C|A|A|C|C|G|C|G|A|A|T|A|T|T|-|-|A|T|T|T|G|G|C|A|C|T|C|C|C|G|T|T|T|
| SD-3.8 | 1/137 |A|C|T|C|T|A|A|G|T|G|-|A|A|A|C|T|T|T|-|C|-|C|G|T|T|C|G|C|T|G|T|T|A|C|G|T|C|A|G|A|T|G|A|G|C|C|G|C|A|C|T|T|T|C|C|A|A|C|C|G|C|G|A|A|T|A|T|T|-|-|A|T|T|T|G|G|C|G|C|T|C|C|C|G|T|T|T|
| SD-3.9 | 1/137 |A|C|T|C|T|A|A|G|T|G|-|A|A|A|C|T|T|T|-|C|-|C|G|T|C|C|G|C|T|G|T|T|A|C|G|T|C|A|G|G|T|G|A|G|C|C|G|C|A|C|T|T|T|C|C|A|A|C|C|G|C|G|A|A|T|A|T|T|-|-|A|T|T|T|G|G|C|G|C|T|C|C|C|G|T|T|T|
| SD-3.10 | 1/137 |A|C|T|C|T|A|A|G|C|G|-|A|A|A|C|T|T|T|T|C|G|T|G|T|T|C|G|C|T|G|T|T|A|C|G|T|C|A|T|G|T|G|A|G|C|C|G|C|A|C|A|T|T|C|C|A|A|C|C|G|C|G|A|A|T|A|T|T|-|-|A|T|T|T|G|G|C|G|C|T|C|C|C|G|T|T|T|
| SD-3.11 | 1/137 |A|C|T|C|T|A|A|G|C|G|C|A|A|-|C|T|T|T|T|C|G|C|G|T|T|C|G|C|T|G|T|T|A|C|G|T|C|A|G|G|T|G|A|G|C|C|G|C|A|C|A|T|T|C|C|A|A|C|C|G|A|G|A|A|T|A|T|T|-|-|A|T|T|T|G|G|C|G|C|T|C|C|C|G|T|T|T|
| SD-3.12 | 1/137 |A|C|T|C|T|A|A|G|C|G|C|A|A|-|C|T|T|T|T|C|G|C|G|T|T|C|G|C|T|G|T|T|A|C|G|T|C|A|G|G|T|G|A|G|C|C|G|T|A|C|A|T|T|C|C|A|A|C|C|G|C|G|A|A|T|A|T|T|-|-|A|T|T|T|G|G|C|G|C|T|C|C|C|G|T|T|T|
| SD-3.13 | 1/137 |A|C|T|C|T|A|A|G|T|G|-|A|A|A|C|T|T|T|-|C|-|C|G|T|T|C|G|C|T|G|T|T|A|C|G|T|C|A|G|G|T|G|A|G|C|C|G|C|A|C|A|T|T|C|C|A|A|C|C|G|C|G|A|A|T|A|T|T|-|-|A|T|T|T|G|G|C|G|C|T|C|C|C|G|T|T|T|
| SD-3.14 | 1/137 |A|C|T|C|T|A|A|G|T|G|-|A|A|A|C|T|T|T|-|C|-|C|G|T|T|C|G|C|T|G|T|T|A|C|G|T|C|A|G|G|T|G|A|G|C|C|G|C|A|C|T|T|T|C|C|A|A|C|C|G|C|G|A|A|T|A|T|T|-|-|A|T|T|T|G|G|C|G|C|C|C|C|C|G|T|T|T|
| SD-3.15 | 1/137 |A|C|T|C|T|A|A|G|C|G|-|A|A|A|C|T|T|T|T|C|G|C|G|T|T|C|G|C|T|G|T|T|A|C|G|T|C|A|T|G|T|G|A|G|C|C|G|C|G|C|A|T|T|C|C|A|A|C|C|G|C|G|A|A|T|A|T|T|-|-|A|T|T|T|G|G|C|G|C|T|C|C|C|G|T|T|T|
| SD-3.16 | 1/137 |A|C|T|C|T|A|A|G|C|G|C|A|A|-|C|T|T|T|T|C|G|C|G|T|T|C|G|C|T|A|T|T|A|C|G|T|C|A|G|G|T|G|A|G|C|C|G|C|A|C|A|T|T|C|C|A|A|C|C|G|C|G|A|A|T|A|T|T|-|-|A|T|T|T|G|G|C|G|C|T|C|C|C|G|T|T|T|
| SD-3.17 | 1/137 |A|C|T|C|T|A|A|G|T|G|-|A|A|A|C|T|T|T|-|C|-|C|G|T|T|C|G|C|T|G|T|T|A|C|G|T|C|A|G|G|T|G|A|T|C|C|G|C|A|C|T|T|T|C|C|A|A|C|C|G|C|G|A|A|T|A|T|T|-|-|A|T|T|T|G|G|C|G|C|T|C|C|C|G|T|T|T|
| SD-3.18 | 1/137 |A|C|T|C|T|A|A|G|C|G|-|A|A|G|C|T|T|T|T|C|G|C|G|T|T|C|G|C|T|G|T|T|A|C|G|T|C|A|T|G|C|G|A|G|C|C|G|C|A|C|T|T|T|C|C|A|A|C|C|G|C|G|A|A|T|A|T|T|-|-|A|T|T|T|G|G|C|G|C|T|C|C|C|G|T|T|T|
| SD-3.19 | 1/137 |A|C|T|C|T|A|A|G|C|G|-|A|A|A|C|T|T|T|T|C|G|C|G|T|T|C|G|C|T|G|T|T|A|C|G|T|C|A|T|G|T|G|A|G|C|C|G|C|A|C|A|T|T|C|C|A|A|C|C|G|C|G|A|A|T|A|T|T|-|-|A|T|T|T|G|G|C|G|C|C|C|C|C|G|T|T|T|
| SD-3.20 | 1/137 |A|C|T|C|T|A|G|G|T|G|-|A|A|A|C|T|T|T|-|C|-|C|G|T|T|C|G|C|T|G|T|T|A|C|G|T|C|A|G|G|T|G|A|G|C|C|G|C|A|C|T|T|T|C|C|A|A|C|C|G|C|G|A|A|T|A|T|T|-|-|A|T|T|T|G|G|C|G|C|T|C|C|C|G|T|T|T|
| SD-4.1 | 1/137 |A|C|T|C|T|A|A|G|C|G|C|A|A|-|C|T|T|T|T|C|G|C|G|T|T|C|G|C|T|G|T|T|A|C|G|T|C|A|G|G|T|G|A|G|C|C|G|C|A|T|A|T|C|C|T|A|A|C|C|G|C|G|A|A|T|A|A|C|C|T|A|T|T|T|G|G|C|G|C|T|C|C|A|G|T|T|T|
| SD-4.2 | 12/137 |A|C|T|C|T|A|A|G|C|G|-|A|A|A|C|T|T|T|-|C|-|C|G|T|T|C|G|C|T|G|T|T|A|C|G|T|C|A|G|G|T|G|A|G|C|C|G|C|A|T|A|T|C|C|T|A|A|C|C|G|C|G|A|A|T|A|A|C|C|T|A|T|T|T|G|G|C|G|C|T|C|C|A|G|T|T|C|
| SD-4.3 | 1/137 |A|C|T|C|C|A|A|-|-|-|T|C|G|-|-|T|T|T|T|C|G|C|G|T|T|C|G|C|T|G|T|T|A|C|G|T|C|A|G|G|T|G|A|G|C|C|G|C|A|C|A|T|T|C|C|A|A|C|C|G|C|G|A|A|T|A|T|T|-|-|A|T|T|T|A|G|C|G|C|T|C|C|A|G|T|T|C|
| SD-4.4 | 1/137 |A|C|T|C|T|A|A|G|C|G|-|A|A|A|C|T|T|T|-|C|-|C|G|T|T|C|G|C|T|G|T|T|A|C|G|T|C|A|G|G|T|G|A|G|C|C|G|C|A|C|A|T|T|C|C|A|A|C|T|G|C|G|A|A|T|A|T|T|-|-|A|T|T|T|G|G|C|G|C|T|C|T|C|G|T|T|T|
| SD-4.5 | 1/137 |A|C|T|C|T|A|A|G|C|G|T|A|A|A|C|T|T|T|-|C|-|C|G|T|T|C|G|T|T|G|T|T|A|C|G|T|C|A|G|G|T|G|A|G|C|C|G|C|A|C|A|T|T|C|C|A|A|C|C|G|C|G|A|A|T|A|T|T|-|-|A|T|T|T|G|G|C|G|C|T|C|T|C|G|T|T|T|
| SD-4.6 | 1/137 |A|C|T|C|T|A|A|G|C|G|-|A|A|A|C|T|T|T|-|C|-|C|G|T|T|C|G|C|T|G|T|T|A|C|G|A|C|A|G|G|T|G|A|G|C|C|G|C|A|C|A|T|T|C|C|A|A|C|C|G|C|G|A|A|T|A|T|T|-|-|A|T|T|T|G|G|C|G|C|T|C|T|C|G|T|T|C|
| SD-4.7 | 1/137 |A|C|T|C|T|A|A|-|-|-|T|C|A|-|-|C|T|T|T|C|G|C|G|T|T|C|G|C|T|G|T|T|A|C|G|T|C|A|G|G|T|G|A|G|C|C|G|C|A|C|A|T|T|C|C|A|A|C|C|G|C|G|A|A|T|A|T|T|-|-|A|T|T|T|G|A|C|G|C|T|C|C|C|G|T|T|T|
| SD-4.8 | 1/137 |A|C|T|C|T|A|A|-|-|-|T|C|A|-|-|T|T|T|T|C|G|C|G|T|T|C|G|C|T|G|T|T|A|C|G|T|C|A|G|G|T|G|A|G|C|C|G|C|A|T|A|T|C|C|T|A|A|C|C|G|C|G|A|A|T|A|A|C|C|T|A|T|T|T|G|G|C|G|C|T|C|C|A|G|T|T|C|
| SD-4.9 | 1/137 |A|C|T|C|T|A|A|G|C|G|-|A|A|A|C|T|T|T|-|C|-|C|G|T|T|C|G|C|T|G|T|T|A|C|G|T|C|A|G|G|T|G|A|G|C|C|G|C|A|C|A|T|T|C|C|A|A|C|C|G|C|G|A|A|T|A|T|T|-|-|A|T|C|T|G|G|C|G|C|T|C|T|C|G|T|T|T|
| SD-4.10 | 1/137 |A|C|T|C|T|G|A|-|-|-|T|C|A|-|-|T|T|T|T|C|G|C|G|T|T|C|G|C|T|G|T|C|A|C|G|T|C|A|G|G|T|G|A|G|C|C|G|C|A|C|A|T|T|C|C|A|A|C|C|G|C|G|A|A|T|A|T|T|-|-|A|T|T|T|G|G|C|G|C|T|C|C|C|G|T|T|T|
| SD-4.11 | 1/137 |A|C|T|C|T|A|A|-|-|-|T|C|A|-|-|T|T|T|-|C|-|C|G|T|T|C|G|C|C|G|T|T|A|C|G|T|C|A|G|G|T|G|A|G|C|C|G|C|A|C|A|T|T|C|C|A|A|C|C|G|C|G|A|A|T|A|T|T|-|-|A|T|T|T|G|G|C|G|T|T|C|T|C|G|T|T|T|

| | | |
|---|---|---|
| SD-4.12 | 1/137 | ACTCTAAGCG-AAACTTT-C-CGTTCGCTGTTACGTCAGGTGAGCCACACATTCCAACCGCGAATATT--ATTTGGCGCTCTCGTTT |
| SD-4.13 | 1/137 | ACCCTAAGCGCAA-CTTTTTGCGTTCGCTGTTACGTCAGGTGAGCCGCACATTCCAACCGCGAATATT--ATTTGGCGCTCTCGTTT |
| SD-4.14 | 1/137 | ACTCTAA---TCA--TTTTCGCGTTCGCTGTTACGTCAGGTGAGCCGCACATTCCAACCGCGAATATT--ATTCGGCGCTCCCGTTT |
| SD-4.15 | 1/137 | ACTCTAAGCGCAA-CTTTTCGCGTTCGCTGTTACGTCAGGTGAGCCGCACATTCCAACCGCGAATGTT--ATTTGGCGCTCCCGTTT |
| SD-4.16 | 1/137 | ACTCTAAGCG-AAACTTT-C-CATTCGCTGTTACGTCAGGTGAGCCGCACATTCCAACCGCGAATATT--ATTTGGCGCTCTCGTTT |
| SD-4.17 | 1/137 | ACTCTAAGCG-AAACTTT-C-CGTTCGCTGTTACGTCAGGTGAGCCGCACATTCCAACCGCGAATATT--ATTTGGCGCTCCCGTTT |
| SD* | 6/137 | ACTCTAAGCG-AAACTTTTCGCGTTCGCTGTTACGTCATGTGAGCCGCACATTCCAACCGCGAATATT--ATTTGGCGCTCCCGTTT |
| SD** | 3/137 | ACTCTAAGCGTAAACTTT-C-CGCTCGCTGTTACGTCAGGTGAGCCGCACATTCCAACCGCGAATATT--ATTTGGCGCTCCCGTTT |
| SD*** | 15/137 | ACTCTAAGCG-AAACTTT-C-CGTTCGCTGTTACGTCAGGTGAGCCGCACATTCCAACCGCGAATATT--ATTTGGCGCTCTCGTTT |
| SD**** | 14/137 | ACTCTAAGCGCAA-CTTTTCGCGTTCGCTGTTACGTCAGGTGAGCCGCACATTCCAACCGCGAATATT--ATTTGGCGCTCCCGTTT |

*Shared haplotype

*Scirtothrips dorsalis* - CO1

| | No. of clone/Total clone | Nucleotide Position | | | | | | | | | | | | | | | | | | | | | | | | | | | | | | | | | | | | | | | | |
|---|---|---|---|---|---|---|---|---|---|---|---|---|---|---|---|---|---|---|---|---|---|---|---|---|---|---|---|---|---|---|---|---|---|---|---|---|---|---|---|---|---|---|
| Specimen | Ratio | 22 | 24 | 31 | 34 | 40 | 62 | 81 | 88 | 120 | 142 | 160 | 193 | 195 | 214 | 236 | 241 | 249 | 279 | 293 | 309 | 326 | 331 | 332 | 336 | 343 | 375 | 406 | 432 | 435 | 467 | 475 | 491 | 497 | 519 | 545 | 556 | 576 | 578 | 601 | 613 | 616 | 628 |
| SD-1.1 | 1/132 | G | T | T | T | T | A | T | A | G | C | T | A | A | A | T | T | A | C | A | A | A | A | A | T | A | C | T | C | T | A | T | A | T | T | T | A | T | T | T | T | A | T |
| SD-1.2 | 1/132 | G | T | T | T | T | A | T | A | A | T | T | A | A | A | T | T | A | C | A | A | A | A | A | T | A | C | T | C | T | A | T | G | T | T | T | A | T | T | C | T | A | T |
| SD-1.3 | 1/132 | G | T | T | T | T | A | T | G | A | T | T | A | A | A | T | T | A | C | A | A | A | A | A | T | A | C | T | C | T | A | T | A | T | T | T | A | T | T | T | C | A | T |
| SD-2.1 | 1/132 | G | T | T | C | T | G | T | A | A | T | T | A | A | A | T | T | A | C | A | A | A | A | A | T | A | C | T | C | T | A | T | A | T | T | T | A | T | T | T | T | A | T |
| SD-2.2 | 1/132 | G | T | T | T | T | A | T | A | A | T | T | A | G | A | T | T | A | C | A | G | A | A | A | T | A | C | T | C | T | A | T | A | T | T | T | A | T | T | T | T | A | T |
| SD-2.3 | 1/132 | G | T | T | T | C | A | T | A | A | T | T | A | A | A | T | T | A | C | A | A | A | G | A | T | A | C | T | C | T | A | T | A | T | T | T | A | T | T | T | T | A | T |
| SD-2.4 | 1/132 | G | T | T | T | T | A | T | A | A | T | T | A | A | A | C | T | A | C | A | A | A | A | A | T | A | C | T | C | T | A | T | A | T | T | T | A | T | C | T | T | A | T |
| SD-2.5 | 1/132 | G | T | T | T | T | A | T | A | A | T | T | A | A | A | T | T | A | C | A | A | A | A | A | T | A | C | T | C | T | A | C | A | T | T | T | A | T | T | T | T | A | T |
| SD-2.6 | 1/132 | G | C | T | T | T | A | T | A | A | T | T | A | A | A | T | T | A | C | A | A | A | A | A | T | A | C | T | C | C | A | T | A | T | T | T | A | T | T | T | T | G | T |
| SD-2.7 | 1/132 | G | T | T | T | T | A | T | A | A | T | T | A | A | A | T | T | A | C | A | A | A | A | A | G | A | T | T | C | T | G | T | A | T | T | T | A | T | T | T | T | A | T |
| SD-2.8 | 1/132 | A | T | T | T | T | A | T | A | A | T | T | A | A | A | T | T | A | C | A | A | A | A | A | T | A | C | T | C | T | A | T | A | C | T | T | A | T | T | T | T | A | T |
| SD-2.9 | 1/132 | G | T | T | T | T | A | T | A | A | T | T | G | A | A | T | C | A | C | A | A | A | A | A | T | A | C | T | T | T | A | T | A | T | T | T | A | T | T | T | T | A | T |
| SD-2.10 | 1/132 | G | T | T | T | T | A | T | A | A | T | T | A | A | A | T | T | A | C | G | A | A | A | A | T | A | C | T | C | T | A | A | A | T | C | T | A | T | T | T | T | A | T |
| SD-3.1 | 1/132 | G | T | C | T | T | A | T | A | A | T | T | A | A | A | T | T | A | C | A | A | A | A | G | T | A | C | T | C | T | A | T | A | T | T | T | A | T | T | T | T | A | T |
| SD-3.2 | 1/132 | G | T | T | T | T | A | T | A | A | T | T | A | A | A | T | T | G | A | A | A | A | A | A | T | A | C | T | C | T | A | T | A | T | T | T | A | T | T | T | T | A | T |
| SD-3.3 | 1/132 | G | T | T | T | T | A | T | A | A | T | T | A | A | G | T | T | A | C | A | A | A | A | A | T | A | C | – | C | T | A | T | A | T | T | T | A | T | T | T | T | A | T |
| SD-4.1 | 1/132 | G | T | T | T | T | A | T | A | A | T | T | A | A | A | T | T | A | C | A | A | A | A | A | T | A | C | T | C | T | A | T | A | T | T | T | G | T | T | T | T | A | T |
| SD-4.2 | 1/132 | G | T | T | T | T | A | C | A | A | T | T | A | A | A | T | T | A | C | A | A | A | A | A | T | A | C | T | C | T | A | T | A | T | T | T | A | T | T | T | T | A | C |
| SD-4.3 | 1/132 | G | T | T | T | T | A | T | A | A | T | – | A | A | A | T | T | A | C | A | A | A | A | A | T | A | C | T | C | T | A | T | A | T | T | T | G | T | T | T | T | A | T |
| SD-4.4 | 1/132 | G | T | T | T | T | A | T | A | A | T | T | A | A | A | C | T | A | C | A | A | G | A | A | T | A | C | T | C | T | A | T | A | T | T | C | A | T | T | T | T | A | T |
| SD* | 110/132 | G | T | T | T | T | A | T | A | A | T | T | A | A | A | T | T | A | C | A | A | A | A | A | T | A | C | T | C | T | A | T | A | T | T | T | A | T | T | T | T | A | T |

*Shared haplotype

*Thrips palmi* - ITS

No.of clone/Total Clone — Nucleotide Position

| Specimen | Ratio | 1 | 17 | 51 | 56 | 68 | 79 | 80 | 97 | 105 | 108 | 118 | 121 | 128 | 130 | 139 | 144 | 153 | 171 | 174 | 192 | 204 | 208 | 212 | 222 | 231 | 249 | 250 | 255 | 260 | 261 | 262 | 267 | 278 | 280 | 289 | 302 | 304 | 305 | 310 | 311 | 320 | 327 | 331 | 334 | 351 | 352 | 355 | 359 | 376 | 387 | 391 | 394 | 398 | 399 | 400 | 408 | 417 | 421 | 423 | 430 | 431 | 432 | 436 | 457 | 468 | 481 | 488 | 490 | 495 | 496 | 498 | 500 | 503 | 513 | 515 | 526 | 537 | 554 | 562 |
|---|---|---|---|---|---|---|---|---|---|---|---|---|---|---|---|---|---|---|---|---|---|---|---|---|---|---|---|---|---|---|---|---|---|---|---|---|---|---|---|---|---|---|---|---|---|---|---|---|---|---|---|---|---|---|---|---|---|---|---|---|---|---|---|---|---|---|---|---|---|---|---|---|---|---|---|---|---|---|---|---|
| TP-1.1 | 1/149 | A | A | A | T | G | A | T | T | C | T | T | A | A | T | T | A | A | A | T | G | G | T | T | G | A | T | T | G | A | C | C | C | A | A | T | C | A | T | C | T | G | G | C | T | T | G | G | T | T | T | G | G | A | T | C | T | G | C | A | C | G | T | T | A | G | T | T | T | T | T | T | T | A | C | C | A | C | T | G |
| TP-1.2 | 1/149 | A | A | A | T | G | T | C | T | C | T | T | A | A | T | T | A | A | A | T | G | A | T | T | G | A | T | T | G | A | C | C | C | A | A | T | C | A | T | C | T | G | G | C | T | T | G | G | T | T | T | G | G | A | T | C | T | G | C | A | T | G | C | C | A | G | T | T | T | T | T | T | T | A | C | C | A | C | T | G |
| TP-1.3 | 1/149 | A | A | A | - | G | A | T | T | C | T | T | A | A | T | T | A | A | A | T | G | A | T | T | G | A | - | - | G | A | C | C | C | A | A | C | C | A | T | C | T | G | G | C | T | G | G | G | T | T | T | G | G | A | T | C | T | G | C | A | T | G | C | C | A | G | T | T | T | T | T | T | T | A | C | C | A | C | T | G |
| TP-1.4 | 1/149 | A | A | A | T | G | A | T | T | C | T | T | A | A | T | T | A | A | A | T | G | A | T | T | G | A | T | T | G | A | C | C | T | A | A | T | C | A | T | C | T | G | G | T | T | G | G | G | T | T | T | G | G | A | T | C | T | G | C | A | T | G | T | C | A | G | T | T | T | T | T | T | T | A | T | T | G | C | T | G |
| TP-1.5 | 1/149 | A | A | A | T | G | A | T | T | C | T | T | A | A | T | T | A | A | A | T | G | A | T | T | G | A | T | T | C | A | C | C | C | A | A | T | C | A | T | C | T | G | G | T | T | G | G | G | T | T | T | A | G | A | T | C | T | G | C | A | T | G | C | C | A | G | T | T | T | T | T | T | T | A | C | C | C | C | T | G |
| TP-1.6 | 1/149 | A | A | A | T | G | T | C | T | C | T | T | A | A | T | T | A | A | A | T | G | A | T | T | G | A | T | T | G | A | C | C | C | A | A | T | C | A | T | C | T | G | G | C | T | T | G | G | T | T | T | A | G | A | T | C | T | G | C | A | T | G | C | C | A | G | T | T | T | T | T | T | T | A | C | C | C | C | T | G |
| TP-1.7 | 2/149 | A | A | A | T | G | A | T | T | C | T | T | A | A | T | T | A | A | A | T | G | A | T | T | G | A | T | T | C | A | C | C | C | A | A | C | C | A | T | T | T | G | G | T | T | G | G | G | T | T | T | G | G | A | T | C | T | G | C | A | T | G | C | C | A | G | T | T | T | T | T | T | T | A | T | T | G | C | T | G |
| TP-1.8 | 1/149 | A | A | A | T | G | A | T | T | C | T | T | A | A | T | T | A | A | A | T | G | A | T | T | G | A | T | T | G | A | C | C | C | A | A | T | C | A | T | C | T | G | G | T | T | G | G | A | T | T | T | A | G | A | T | C | T | G | C | A | T | G | C | C | A | G | T | T | T | T | T | T | T | A | C | C | C | C | T | G |
| TP-1.9 | 1/149 | A | A | A | T | G | A | T | T | C | T | T | A | A | T | T | A | A | A | T | G | A | T | T | G | A | T | T | C | A | T | C | C | A | A | C | C | A | T | T | T | G | G | T | T | G | G | G | T | T | T | G | G | A | T | C | T | G | C | A | T | G | C | C | A | G | T | T | T | T | T | T | T | A | C | C | C | C | T | G |
| TP-1.10 | 1/149 | A | A | A | T | G | A | T | T | C | T | T | A | A | T | T | A | A | A | T | G | A | T | T | G | A | - | - | G | A | C | C | C | A | A | C | C | A | T | C | T | G | G | C | T | G | G | G | T | C | T | G | G | A | T | C | T | G | C | A | T | G | C | C | A | G | T | T | T | T | T | T | T | A | C | C | C | C | C | G |
| TP-1.11 | 1/149 | A | A | A | T | G | A | T | T | C | T | T | A | A | T | A | A | A | A | T | G | A | T | T | G | A | T | T | G | A | C | C | C | A | A | T | C | A | T | C | T | G | G | C | T | G | G | G | T | T | T | A | G | A | T | C | T | G | C | A | T | G | C | C | A | G | T | T | T | T | T | T | T | A | T | T | G | T | T | G |
| TP-1.12 | 1/149 | A | A | A | T | G | T | C | T | C | T | T | A | A | T | T | A | A | A | T | G | A | T | T | G | A | T | T | G | A | C | C | C | A | A | T | C | A | T | C | T | G | G | T | T | G | G | G | T | T | T | G | G | A | T | C | T | G | C | A | T | G | C | C | A | G | T | T | T | T | T | T | T | A | C | C | C | C | T | A |
| TP-1.13 | 1/149 | A | A | A | T | G | T | C | T | C | T | T | A | A | T | T | A | A | A | C | G | A | T | T | G | A | T | T | G | A | C | C | C | A | A | T | C | A | T | C | T | G | G | T | T | G | G | G | T | T | T | A | G | A | T | C | T | G | C | A | T | G | C | C | A | G | T | T | T | T | T | T | T | A | C | C | C | C | T | G |
| TP-1.14 | 1/149 | A | A | A | T | G | A | T | T | C | T | T | A | A | T | T | A | A | A | T | G | A | T | T | G | A | T | T | C | A | C | C | C | A | A | C | C | A | T | T | T | G | G | T | T | G | G | G | T | T | T | G | G | A | T | C | T | G | C | A | T | G | C | C | A | G | T | T | T | T | T | T | T | A | C | C | C | C | T | G |
| TP-1.15 | 1/149 | A | A | A | T | G | A | T | T | C | T | T | A | A | T | T | A | A | A | T | G | A | T | T | G | A | T | T | G | A | C | C | C | A | A | T | C | A | T | C | T | G | G | T | T | T | G | G | T | T | T | G | G | A | T | C | T | G | C | A | C | G | T | T | A | G | T | T | T | T | T | T | T | A | C | C | C | C | T | G |
| TP-1.16 | 1/149 | A | A | A | T | G | A | T | T | C | T | T | A | A | T | T | A | A | A | T | G | A | T | T | G | A | - | - | G | A | C | C | C | A | A | C | C | A | T | C | T | G | G | T | T | G | G | G | T | T | T | G | G | A | T | C | T | G | C | A | T | G | C | C | A | G | T | T | T | T | T | T | T | A | C | C | C | C | T | G |
| TP-1.17 | 1/149 | A | A | A | T | G | A | T | T | C | T | T | A | A | T | T | A | A | A | T | G | A | T | T | G | A | - | - | G | A | C | C | C | A | A | T | C | A | T | C | T | G | G | C | T | G | G | G | T | T | T | G | G | A | T | C | T | G | C | A | T | G | T | C | A | G | T | T | T | T | T | T | T | A | T | T | G | T | T | G |
| TP-2.1 | 1/149 | A | A | G | T | G | A | T | T | C | T | T | A | A | T | T | A | A | A | T | G | A | T | T | G | A | T | T | G | A | C | C | C | A | A | C | C | A | T | T | T | G | G | T | T | G | G | G | T | T | T | G | G | A | T | C | T | G | C | A | T | G | C | C | A | G | T | T | T | T | T | T | T | A | C | C | A | C | T | G |
| TP-2.2 | 1/149 | A | A | A | T | G | A | T | T | C | T | T | A | A | T | T | A | A | A | T | G | A | T | T | G | A | T | T | G | A | C | C | C | A | A | T | C | A | T | C | T | G | G | T | T | G | G | G | T | T | T | G | G | A | T | C | T | G | C | A | T | G | T | C | A | G | T | T | T | T | T | T | T | A | C | C | A | C | T | G |
| TP-2.3 | 1/149 | A | A | A | T | G | T | C | T | C | T | T | A | A | T | T | G | A | A | T | G | A | T | T | G | A | T | T | G | A | C | C | C | A | A | T | C | A | T | C | T | G | G | C | T | T | G | G | T | T | T | G | G | A | T | C | T | G | C | A | T | G | C | C | G | G | T | T | T | T | T | T | T | A | C | C | A | C | T | G |
| TP-2.4 | 1/149 | A | A | A | T | G | T | C | T | C | T | T | A | A | T | T | A | A | A | T | G | A | T | T | G | A | T | T | G | A | C | C | C | A | A | T | T | A | T | C | T | G | G | T | T | G | G | G | T | T | T | G | G | A | T | C | T | G | C | A | T | G | C | C | A | G | T | T | T | T | T | T | T | A | C | C | A | C | T | G |
| TP-2.5 | 1/149 | A | A | A | T | G | A | T | T | C | T | T | A | A | T | T | A | A | A | T | G | A | T | T | G | A | T | T | G | A | C | C | C | A | A | T | C | A | T | C | T | G | G | T | T | G | G | G | T | T | T | A | G | A | T | C | T | G | C | G | T | G | C | C | A | G | T | T | T | T | T | T | T | A | C | C | A | C | T | G |
| TP-2.6 | 1/149 | A | A | A | T | G | A | T | T | C | T | T | A | A | T | C | A | A | A | T | G | A | T | T | G | A | - | - | G | A | C | C | C | A | A | C | C | A | T | C | T | G | G | C | T | G | G | G | T | T | T | G | G | A | T | C | T | A | C | A | T | G | C | C | A | G | T | T | T | T | T | T | T | A | C | C | A | C | T | G |
| TP-2.7 | 1/149 | A | A | A | T | A | A | T | T | C | T | T | A | A | T | T | A | A | A | T | G | A | T | T | G | A | T | T | G | A | C | C | C | A | A | T | C | A | T | C | T | G | G | T | T | G | G | G | T | T | T | A | G | A | T | C | T | G | C | A | T | G | C | C | A | G | T | T | T | T | T | T | T | A | C | C | A | C | T | G |
| TP-2.8 | 1/149 | A | A | A | T | G | A | T | T | C | T | T | A | A | T | T | A | A | A | T | G | A | T | T | G | A | T | T | G | A | C | C | C | A | A | T | C | A | T | C | T | G | G | T | T | G | G | G | T | T | T | G | G | A | T | C | T | G | C | A | T | G | T | T | A | G | T | T | T | T | T | T | T | A | C | C | A | C | T | G |
| TP-2.9 | 1/149 | A | A | A | T | G | A | T | T | C | T | T | A | A | T | T | A | A | A | T | G | A | T | T | G | A | T | T | G | A | C | C | C | A | A | T | C | A | T | C | T | G | G | T | T | G | G | G | T | T | T | A | G | A | T | C | T | G | C | A | T | A | C | C | A | G | T | T | T | T | T | T | T | A | C | C | A | C | T | G |
| TP-2.10 | 1/149 | A | A | A | T | G | A | T | T | C | T | T | A | A | T | T | A | A | A | T | G | A | T | T | G | A | T | T | G | A | C | C | C | A | A | T | C | A | T | C | T | G | G | C | T | T | G | G | T | T | C | G | G | A | T | C | T | G | C | A | C | G | T | T | A | G | T | T | T | T | T | T | T | A | C | C | A | C | T | G |
| TP-2.11 | 1/149 | A | A | A | T | G | T | C | T | C | T | T | A | A | T | T | A | A | A | T | G | A | T | T | G | A | T | T | G | A | C | C | C | A | A | T | C | A | T | C | T | G | G | C | T | T | G | G | T | T | T | G | A | A | T | C | T | G | C | A | C | G | T | T | A | G | T | T | T | T | T | T | T | A | C | C | A | C | T | G |
| TP-2.12 | 1/149 | A | A | A | T | G | A | T | T | C | T | T | A | A | T | T | A | A | A | T | G | A | T | T | G | A | T | T | G | A | C | C | C | A | A | T | C | A | T | C | T | G | G | T | T | G | G | G | T | T | T | G | G | A | T | C | T | G | C | A | C | G | T | T | A | G | T | T | T | T | T | T | T | A | C | C | A | C | T | G |
| TP-2.13 | 1/149 | A | A | A | T | G | T | C | T | C | T | T | A | A | T | T | A | A | A | T | G | A | T | T | G | A | T | T | G | A | C | C | C | A | A | T | C | A | T | C | T | G | G | T | T | G | G | G | T | T | T | G | G | A | T | C | T | G | C | A | T | G | C | C | A | G | T | T | T | T | T | T | C | A | T | T | G | T | T | G |
| TP-2.14 | 1/149 | A | A | A | T | G | A | T | T | C | T | T | A | A | T | T | A | A | A | T | G | A | T | T | G | A | T | T | G | A | C | C | C | A | A | T | C | A | T | C | T | G | G | T | T | G | G | G | T | T | T | A | G | A | T | C | T | G | C | A | C | G | T | T | A | G | T | T | T | T | T | T | T | A | C | C | A | C | T | G |
| TP-3.1 | 1/149 | A | A | A | T | G | A | T | T | C | T | T | A | A | T | T | A | A | A | T | G | A | T | T | G | A | - | - | G | A | C | C | C | A | A | C | C | A | T | C | C | G | G | C | T | G | G | G | T | T | T | G | G | A | T | C | T | G | C | A | T | G | T | C | A | G | T | T | T | T | T | T | T | A | C | C | A | C | T | G |
| TP-3.2 | 7/149 | A | A | A | T | G | T | C | T | C | T | T | A | A | T | T | A | A | A | T | G | A | T | T | G | A | T | T | G | A | C | C | C | A | A | T | C | A | T | C | T | G | G | T | T | G | G | G | T | T | T | G | G | A | T | C | A | G | C | A | T | G | C | T | A | G | T | T | T | T | T | T | T | A | C | C | A | C | T | G |
| TP-3.3 | 1/149 | A | A | A | T | G | A | T | T | C | T | T | A | A | T | T | A | A | A | T | G | A | T | T | G | A | T | T | G | A | C | C | C | A | A | T | C | A | T | C | T | G | G | T | T | G | A | G | T | T | T | G | G | A | T | C | T | G | C | A | T | G | C | C | A | G | T | T | T | T | T | T | T | A | C | C | A | C | T | G |
| TP-3.4 | 1/149 | A | A | A | T | G | T | C | T | C | T | T | A | A | T | T | A | A | A | T | G | A | T | T | G | A | T | T | G | A | C | C | C | A | A | T | C | G | T | C | T | G | G | C | T | T | G | G | T | T | T | G | G | A | T | C | T | G | C | A | T | G | T | C | A | G | T | T | T | C | T | T | T | A | T | T | G | T | T | G |
| TP-3.5 | 1/149 | G | A | A | T | G | A | T | T | C | T | T | A | A | T | T | A | A | A | T | G | A | T | T | G | A | - | - | G | A | C | C | C | A | G | C | C | A | T | C | T | G | G | C | T | G | G | G | T | T | T | G | G | A | T | C | T | G | C | A | T | G | T | C | A | G | T | T | T | T | T | T | T | A | T | T | G | T | T | G |
| TP-3.6 | 1/149 | A | A | A | T | G | T | C | T | C | T | T | A | A | T | T | A | A | A | T | G | A | T | T | G | A | T | T | G | A | C | C | C | A | A | T | C | A | T | C | T | G | G | T | T | G | G | G | T | T | T | G | G | A | T | C | T | G | C | A | T | G | T | T | A | G | T | T | T | T | T | T | T | A | C | C | A | C | T | G |
| TP-3.7 | 1/149 | A | A | A | T | G | T | C | T | C | T | T | G | A | T | T | A | A | A | T | G | A | T | T | G | A | T | T | G | A | C | C | C | A | A | T | C | A | T | C | T | G | G | T | T | G | G | G | T | T | T | G | G | A | T | C | A | G | C | A | T | G | C | T | A | G | T | T | T | T | T | T | T | A | C | C | A | C | T | G |
| TP-3.8 | 1/149 | A | A | A | T | G | A | T | T | C | T | T | A | A | T | T | A | A | A | T | G | A | T | T | G | A | T | T | G | A | C | C | C | A | A | T | C | A | T | C | T | G | G | T | T | G | G | G | T | T | T | G | G | A | T | C | A | G | C | A | T | G | C | T | A | G | T | T | T | T | T | C | T | A | C | C | A | C | T | G |
| TP-3.9 | 1/149 | A | A | A | T | G | A | T | T | C | T | T | A | A | T | T | A | A | A | T | G | A | T | T | G | A | - | - | G | A | C | C | C | A | A | C | C | A | T | C | T | G | G | C | T | G | G | G | T | T | T | G | G | A | T | C | T | G | C | A | T | G | T | C | A | G | T | T | T | C | T | T | T | A | T | T | G | C | T | G |
| TP-3.10 | 1/149 | G | A | A | T | G | T | C | T | C | T | T | A | A | T | T | A | A | G | T | G | A | T | T | G | A | - | - | G | A | C | C | C | A | A | C | C | A | T | C | T | G | G | C | T | G | G | G | T | T | T | G | G | A | T | C | T | G | C | A | T | G | T | C | A | G | T | T | T | T | T | T | T | A | C | C | A | C | T | G |
| TP-3.11 | 1/149 | A | A | A | T | G | T | C | T | C | T | T | A | A | T | T | A | A | A | T | G | A | T | T | G | A | T | T | G | A | C | C | C | G | A | T | C | A | T | C | T | G | G | T | T | G | G | G | T | T | T | G | G | A | T | C | T | G | C | A | T | G | C | C | A | G | T | T | T | T | T | T | T | A | C | C | A | C | T | G |
| TP-3.12 | 1/149 | A | A | A | T | G | T | C | T | C | T | T | A | A | T | T | A | A | A | T | A | A | T | T | G | A | T | T | G | A | C | C | C | A | A | T | C | A | T | C | T | G | G | T | T | G | G | G | T | T | T | G | G | G | T | C | T | G | C | A | T | G | C | C | A | G | T | T | T | T | T | T | T | A | C | C | A | C | T | G |
| TP-3.13 | 1/149 | A | A | A | T | G | A | T | T | C | T | T | A | A | T | T | A | A | A | T | G | A | T | T | G | A | - | - | G | A | C | C | C | A | A | C | C | A | T | C | T | G | G | C | T | G | G | G | T | T | T | G | G | A | T | C | T | G | C | A | T | G | T | C | A | G | T | T | T | T | T | T | T | A | T | T | A | C | T | G |
| TP-4.1 | 3/149 | A | A | A | T | G | T | C | T | C | T | T | A | A | T | T | A | A | A | T | G | A | T | T | G | A | T | T | G | A | C | C | C | A | A | T | C | A | T | C | T | T | G | T | T | G | G | G | T | T | T | G | G | A | T | C | T | G | C | A | T | G | C | C | A | G | T | T | T | T | T | T | T | A | C | C | A | C | T | G |
| TP-4.2 | 1/149 | A | A | A | T | G | A | T | T | C | T | T | A | A | T | T | A | A | A | T | G | A | T | T | G | A | - | - | G | A | C | C | C | A | A | C | C | A | T | C | T | G | G | C | T | G | G | G | T | T | T | A | G | A | T | C | T | G | C | A | T | G | C | C | A | G | T | T | T | T | T | T | T | A | C | C | A | C | T | G |
| TP-4.3 | 2/149 | A | A | A | T | G | T | C | T | C | T | T | A | A | T | T | A | A | A | T | G | A | T | T | G | A | T | T | G | A | C | C | C | A | A | T | C | A | T | C | T | G | G | C | T | T | G | G | T | T | T | G | G | A | T | C | T | G | C | A | C | G | T | T | A | G | T | T | T | T | T | T | T | A | C | C | A | T | T | G |
| TP-4.4 | 1/149 | A | A | A | T | G | A | T | T | C | T | T | A | A | T | T | A | A | A | T | G | A | T | T | G | A | T | T | G | A | C | T | T | A | A | T | C | A | T | C | T | G | G | T | T | G | G | G | T | T | T | G | G | A | T | C | T | G | C | A | T | G | C | C | A | G | T | T | T | T | T | T | T | A | C | C | A | T | T | G |
| TP-4.5 | 4/149 | A | A | A | T | G | A | T | T | C | T | T | A | A | T | T | A | A | A | T | G | A | T | T | G | A | T | T | G | A | C | T | C | A | A | T | C | A | T | C | T | G | G | T | T | G | G | G | T | T | T | A | G | A | T | C | T | G | C | A | T | G | C | C | A | G | T | T | T | T | T | T | T | A | C | C | A | C | T | G |
| TP-4.6 | 1/149 | A | A | A | T | G | A | T | T | C | T | T | A | A | T | T | A | G | A | T | G | A | T | T | G | A | - | - | G | A | C | C | C | A | A | C | C | A | T | C | T | G | G | C | T | G | G | G | T | T | T | G | G | A | T | C | T | G | C | A | T | G | T | C | A | G | T | T | T | T | T | T | T | A | T | T | G | T | T | G |
| TP-4.7 | 1/149 | A | A | A | T | G | A | T | T | T | T | T | A | A | T | T | A | A | A | T | G | A | T | T | G | A | T | T | G | A | C | C | C | A | A | T | C | A | T | C | T | G | G | T | T | G | G | G | T | T | T | G | G | A | C | T | T | G | C | A | T | G | C | T | A | G | T | T | T | T | T | T | T | A | T | T | G | T | T | G |
| TP-4.8 | 1/149 | A | A | A | T | G | T | C | T | C | T | T | A | A | T | T | A | A | A | T | G | A | T | C | G | A | T | T | G | A | C | C | C | A | A | T | C | A | T | C | T | T | G | T | T | G | G | G | T | T | T | G | G | A | C | C | T | G | C | A | T | G | C | C | A | G | C | T | C | T | T | T | T | A | C | C | A | C | T | G |
| TP-4.9 | 1/149 | A | A | A | T | G | A | T | T | C | T | T | A | A | T | T | A | A | A | T | G | A | T | T | G | A | T | T | G | A | C | T | C | A | A | T | C | A | T | C | T | G | G | T | T | G | G | G | T | T | T | A | G | A | T | C | T | G | C | A | T | G | C | C | A | G | T | T | T | T | T | T | T | A | T | T | G | T | T | G |
| TP-4.10 | 1/149 | A | A | A | T | G | A | T | T | C | T | T | A | A | T | T | A | A | A | T | G | A | T | T | G | A | T | T | G | A | C | T | C | A | A | T | C | A | T | C | T | G | G | T | T | G | G | G | C | T | T | A | G | A | T | C | T | G | C | A | T | G | C | C | A | G | T | A | T | T | T | T | T | A | C | C | A | C | T | G |
| TP-4.11 | 1/149 | A | A | A | T | G | A | T | T | C | T | T | A | A | T | T | A | A | A | T | G | A | T | T | G | A | T | T | G | A | C | T | C | A | A | T | C | A | T | C | T | G | G | T | T | G | G | G | T | T | T | A | G | A | T | C | T | G | G | A | T | G | C | C | A | G | T | T | T | T | T | T | T | A | C | C | A | C | T | G |
| TP-4.12 | 1/149 | A | G | A | T | G | A | T | T | C | T | T | A | A | T | T | A | A | A | T | G | A | T | T | G | A | T | T | G | A | C | C | C | A | A | T | C | A | T | C | T | G | G | C | T | G | G | G | T | T | T | G | G | A | T | C | T | G | C | A | T | G | C | C | A | G | T | T | T | T | T | T | T | A | C | C | A | C | T | G |
| TP-4.13 | 1/149 | A | A | A | T | G | A | T | T | C | T | T | A | A | T | T | A | A | A | T | G | A | T | T | A | A | T | T | G | A | C | T | C | A | A | T | C | A | T | C | T | G | G | T | T | G | G | G | T | T | T | A | G | A | T | C | T | G | C | A | T | G | C | C | A | G | T | T | T | T | T | T | T | A | C | C | A | C | T | G |
| TP-4.14 | 1/149 | A | A | A | T | G | A | T | T | C | T | T | A | A | T | T | A | A | A | T | G | A | T | T | G | A | T | T | G | A | C | T | C | A | A | T | C | A | T | C | T | G | A | T | T | G | G | G | T | T | T | A | G | A | T | C | T | G | C | A | T | G | C | C | A | G | T | T | T | T | T | T | T | A | C | C | A | T | T | G |

| | | | | | | | | | | | | | | | | | | | | | | | | | | | | | | | | | | | | | | | | | | | | | | | | | | | | | | | | | | | | | | | | | | | | | | | | | | | | | | | | |
|---|---|---|---|---|---|---|---|---|---|---|---|---|---|---|---|---|---|---|---|---|---|---|---|---|---|---|---|---|---|---|---|---|---|---|---|---|---|---|---|---|---|---|---|---|---|---|---|---|---|---|---|---|---|---|---|---|---|---|---|---|---|---|---|---|---|---|---|---|---|---|---|---|---|---|---|---|---|---|---|---|
| TP-4.15 | 1/149 | A | A | A | T | G | T | C | T | C | T | C | A | A | T | T | A | A | A | T | G | A | T | T | G | A | T | T | G | A | C | C | C | A | A | T | C | A | T | C | T | T | G | T | T | G | G | G | T | T | T | G | G | A | T | C | T | G | C | A | T | G | C | C | A | G | T | T | T | T | T | T | T | A | C | C | A | C | T | G |
| TP-4.16 | 1/149 | A | A | A | T | G | T | C | T | C | T | T | A | A | T | T | A | A | A | T | G | A | T | T | G | A | T | T | G | A | C | C | C | A | A | T | C | A | T | C | T | T | G | T | T | G | G | G | T | T | T | G | G | A | T | C | T | G | C | A | T | G | C | C | A | G | T | T | T | T | C | T | T | A | C | C | A | C | T | G |
| TP-4.17 | 1/149 | A | A | A | T | G | T | C | T | C | T | T | A | G | T | T | A | A | A | T | G | A | T | T | G | A | T | T | G | A | C | T | C | A | A | T | C | A | T | C | T | G | G | T | T | G | G | G | T | T | T | A | G | A | T | C | T | G | C | A | T | G | C | C | A | G | T | T | T | T | T | T | T | A | C | C | A | C | T | G |
| TP-4.18 | 1/149 | A | A | A | T | G | A | T | T | C | T | T | A | A | T | T | A | A | A | T | G | A | T | T | G | A | – | – | G | A | C | C | C | A | A | C | C | A | T | C | T | G | G | C | T | G | G | G | T | T | T | G | G | A | T | C | T | G | C | A | T | G | T | C | A | G | T | T | T | T | T | T | T | G | T | T | G | T | T | G |
| TP-4.19 | 1/149 | A | A | A | T | G | A | T | T | C | T | T | A | A | T | T | A | A | A | T | G | A | C | T | G | G | T | T | G | A | C | T | C | A | A | T | C | A | T | C | T | G | G | T | T | G | G | G | T | T | T | A | G | A | T | C | T | G | C | A | T | G | C | C | A | G | T | T | T | T | T | T | T | A | C | C | A | C | T | G |
| TP-4.20 | 1/149 | A | A | A | T | G | A | T | T | C | T | T | A | A | T | T | A | A | A | T | G | A | T | T | G | A | T | T | G | G | C | C | C | A | A | T | C | A | T | C | T | G | G | T | T | G | G | G | T | T | T | G | G | G | T | C | T | G | C | A | T | G | C | C | A | G | T | T | T | T | T | T | T | A | C | C | A | C | T | G |
| TP-4.21 | 1/149 | A | A | A | T | G | T | C | T | T | T | T | A | A | T | T | A | A | A | T | G | A | T | T | G | A | T | T | G | A | C | C | C | A | A | T | C | A | T | C | T | G | G | T | T | G | G | G | T | T | T | G | G | A | T | C | T | G | C | A | T | G | C | C | A | G | T | T | T | T | T | T | T | A | C | C | A | C | T | G |
| TP-4.22 | 1/149 | A | A | A | T | G | T | C | T | C | T | T | A | A | T | T | A | A | A | T | G | A | T | T | A | A | T | T | G | A | C | C | C | A | A | T | C | A | T | C | T | G | G | C | T | T | G | G | T | T | T | G | G | A | T | C | T | G | C | A | C | G | T | T | A | A | T | T | T | T | T | T | T | A | C | C | A | T | T | G |
| TP-4.23 | 1/149 | A | A | A | T | G | T | C | T | C | C | T | A | A | T | T | A | A | A | T | G | A | T | T | G | A | T | T | G | A | C | C | C | A | A | T | C | A | T | C | T | G | G | C | T | T | G | G | T | T | T | G | G | A | T | C | T | G | C | A | C | G | T | T | A | G | T | T | T | T | T | T | T | A | C | C | A | T | T | G |
| TP-4.24 | 1/149 | A | A | A | T | G | A | T | C | C | T | T | A | A | T | T | A | A | A | T | G | A | T | T | G | A | T | T | G | A | C | C | C | A | A | T | C | A | T | C | T | G | G | T | T | G | G | G | T | T | T | G | G | A | C | T | T | G | C | A | T | G | C | T | A | G | T | T | T | T | T | T | T | A | T | T | G | T | T | G |
| TP-4.25 | 1/149 | A | A | A | T | G | A | T | T | C | T | T | A | A | C | T | A | A | A | T | G | A | T | T | G | A | – | – | G | A | C | C | C | A | A | C | C | A | T | C | T | G | G | C | T | G | G | G | T | T | T | G | G | A | T | C | T | G | C | A | T | G | T | C | A | G | T | T | T | T | T | T | T | A | C | C | A | C | T | G |
| TP-4.26 | 1/149 | A | A | A | T | G | A | T | T | C | T | T | A | A | T | T | A | A | A | T | G | A | T | T | G | A | T | T | G | A | C | C | C | A | A | T | C | A | C | C | T | G | G | T | C | G | G | G | T | T | T | G | G | A | T | C | T | G | C | A | T | G | C | C | A | G | T | T | T | T | T | T | T | A | C | C | A | C | T | G |
| TP* | 22/149 | A | A | A | T | G | A | T | T | C | T | T | A | A | T | T | A | A | A | T | G | A | T | T | G | A | T | T | G | A | C | C | C | A | A | T | C | A | T | C | T | G | G | T | T | G | G | G | T | T | T | G | G | A | T | C | T | G | C | A | T | G | C | C | A | G | T | T | T | T | T | T | T | A | C | C | A | C | T | G |
| TP** | 17/149 | A | A | A | T | G | A | T | T | C | T | T | A | A | T | T | A | A | A | T | G | A | T | T | G | A | T | T | G | A | C | C | C | A | A | T | C | A | T | C | T | G | G | T | T | G | G | G | T | T | T | A | G | A | T | C | T | G | C | A | T | G | C | C | A | G | T | T | T | T | T | T | T | A | C | C | A | C | T | G |
| TP*** | 2/149 | A | A | A | T | G | A | T | T | C | T | T | A | A | T | T | A | A | A | T | G | A | T | T | G | A | – | – | G | A | C | C | C | A | A | C | C | A | T | C | T | G | G | C | T | G | G | G | T | T | T | G | G | A | T | C | T | G | C | A | T | G | T | C | A | G | T | T | T | T | T | T | T | A | C | C | C | C | T | G |
| TP**** | 6/149 | A | A | A | T | G | T | C | T | C | T | T | A | A | T | T | A | A | A | T | G | A | T | T | G | A | T | T | G | A | C | C | C | A | A | T | C | A | T | C | T | G | G | C | T | T | G | G | T | T | T | G | G | A | T | C | T | G | C | A | C | G | T | T | A | G | T | T | T | T | T | T | T | A | C | C | A | C | T | G |
| TP***** | 10/149 | A | A | A | T | G | T | C | T | C | T | T | A | A | T | T | A | A | A | T | G | A | T | T | G | A | T | T | G | A | C | C | C | A | A | T | C | A | T | C | T | G | G | T | T | G | G | G | T | T | T | G | G | A | T | C | T | G | C | A | T | G | C | C | A | G | T | T | T | T | T | T | T | A | C | C | A | C | T | G |
| TP****** | 9/149 | A | A | A | T | G | A | T | T | C | T | T | A | A | T | T | A | A | A | T | G | A | T | T | G | A | – | – | G | A | C | C | C | A | A | C | C | A | T | C | T | G | G | C | T | G | G | G | T | T | T | G | G | A | T | C | T | G | C | A | T | G | T | C | A | G | T | T | T | T | T | T | T | A | T | T | G | T | T | G |

*Shared haplotype

***Thrips palmi* -CO1**

| | No.of clone/Total Clone | Nucletide Position | | | | | | | | | | | | | | | | | | | |
|---|---|---|---|---|---|---|---|---|---|---|---|---|---|---|---|---|---|---|---|---|---|
| **Specimen** | **Ratio** | **30** | **44** | **79** | **160** | **187** | **267** | **270** | **280** | **308** | **313** | **328** | **339** | **363** | **386** | **408** | **572** | **578** | **598** | **601** | **643** |
| TP-1.1 | 38/120 | C | T | A | T | T | T | T | T | A | A | A | A | A | A | C | A | T | T | A | C |
| TP-1.2 | 1/120 | C | T | A | T | T | T | T | T | A | A | A | A | A | A | A | A | C | T | A | C |
| TP-1.3 | 1/120 | C | T | A | T | T | T | C | T | G | A | A | A | A | A | C | A | T | T | A | C |
| TP-1.4 | 1/120 | C | T | G | T | T | T | T | T | A | A | A | A | A | A | C | A | T | T | A | T |
| TP-1.5 | 1/120 | T | T | A | T | T | T | T | T | A | A | A | A | A | A | C | G | T | T | A | C |
| TP-2.1 | 1/120 | C | C | A | – | C | T | T | T | A | G | A | A | A | A | C | A | T | C | A | C |
| TP-2.2 | 1/120 | C | C | A | T | C | C | T | T | A | A | A | A | G | A | C | A | T | C | A | C |
| TP-4.1 | 1/120 | C | C | A | T | C | T | T | C | A | A | G | A | A | A | C | A | T | C | A | C |
| TP-4.2 | 1/120 | C | C | A | T | C | T | T | T | A | A | A | G | A | A | C | A | T | C | G | C |
| TP-4.3 | 1/120 | C | C | A | T | C | T | T | T | A | A | A | A | A | G | C | A | T | C | G | C |
| TP* | 73/120 | C | C | A | T | C | T | T | T | A | A | A | A | A | A | C | A | T | C | A | C |

*Shared haplotype

## *Frankliniella occidentalis* -ITS

| No.of clone/Total Clone | | Nucletide Position | | | | | | | | | | | | | | | | | | | | | | | | | |
|---|---|---|---|---|---|---|---|---|---|---|---|---|---|---|---|---|---|---|---|---|---|---|---|---|---|---|---|
| **Specimen** | **Ratio** | **34** | **37** | **70** | **92** | **127** | **149** | **158** | **169** | **173** | **179** | **237** | **240** | **246** | **252** | **258** | **264** | **289** | **291** | **295** | **299** | **313** | **328** | **386** | **387** | **416** | **432** |
| FO-1.1 | 17/105 | A | A | G | A | T | T | C | T | T | G | G | A | C | C | A | T | G | T | T | G | A | T | – | – | G | A |
| FO-1.2 | 1/105 | A | G | G | A | T | T | T | T | T | G | G | A | C | C | A | T | G | T | T | G | A | T | – | – | G | A |
| FO-1.3 | 1/105 | A | A | G | G | T | T | C | T | T | G | G | A | C | C | A | T | G | T | T | G | G | T | – | – | G | A |
| FO-1.4 | 1/105 | A | A | G | A | T | T | C | T | T | G | G | A | C | C | A | T | G | T | T | G | G | C | – | – | G | A |
| FO-2.1 | 1/105 | G | A | G | A | T | T | C | T | C | G | G | A | C | C | A | T | G | T | T | G | G | C | T | T | G | A |
| FO-3.1 | 1/105 | A | A | G | A | C | T | C | T | T | G | A | A | C | C | A | T | G | T | T | G | G | C | T | T | G | A |
| FO-3.2 | 1/105 | A | A | G | A | T | T | C | T | T | G | G | A | C | C | G | T | G | T | T | A | G | C | T | T | G | A |
| FO-3.3 | 1/105 | A | A | G | A | T | T | C | C | T | G | G | A | C | T | A | T | G | T | T | G | G | C | T | T | G | A |
| FO-3.4 | 1/105 | A | A | G | A | T | T | C | T | T | A | G | A | T | C | A | T | G | T | T | G | G | C | T | T | G | A |
| FO-3.5 | 1/105 | A | A | G | A | T | C | C | T | T | G | G | A | C | C | A | C | G | T | T | G | G | C | T | T | T | A |
| FO-3.6 | 1/105 | A | A | G | A | T | T | C | T | T | G | G | A | C | C | A | T | G | T | C | G | G | C | T | T | G | G |
| FO-4.1 | 1/105 | A | A | G | A | T | T | C | T | T | G | G | A | C | C | A | T | T | A | T | G | G | C | T | T | G | A |
| FO-4.2 | 1/105 | A | A | A | A | T | T | C | T | T | G | G | G | C | C | A | T | G | T | T | G | G | C | T | T | G | A |
| FO* | 76/105 | A | A | G | A | T | T | C | T | T | G | G | A | C | C | A | T | G | T | T | G | G | C | T | T | G | A |

*Shared haplotype

### *Frankliniella occidentalis* CO1

| | No.of clone/Total Clone | Nucletide Position | | | | | | | | | | | | | | | | | | | | | | |
|---|---|---|---|---|---|---|---|---|---|---|---|---|---|---|---|---|---|---|---|---|---|---|---|---|
| **Specimen** | **Ratio** | **30** | **31** | **34** | **41** | **48** | **88** | **113** | **114** | **133** | **137** | **158** | **189** | **190** | **216** | **252** | **268** | **297** | **303** | **321** | **352** | **383** | **399** | **414** |
| FO-1.1 | 1/150 | T | T | T | T | T | A | T | C | A | T | T | T | A | T | A | T | C | T | C | A | G | T | T |
| FO-1.2 | 1/150 | T | T | T | T | C | A | T | T | A | T | T | T | A | T | A | T | T | T | C | G | G | T | T |
| FO-1.3 | 1/150 | T | T | T | T | C | A | T | C | A | T | T | T | G | T | A | T | T | T | C | A | G | C | T |
| FO-1.4 | 2/150 | T | T | T | T | C | G | T | C | A | T | T | T | A | T | A | T | T | T | C | A | G | T | T |
| FO-1.5 | 1/150 | C | T | T | T | C | A | T | C | A | T | T | T | A | T | A | T | T | C | C | A | G | T | T |
| FO-2.1 | 2/150 | T | T | T | T | C | A | C | C | A | T | T | T | A | T | A | T | T | T | C | A | G | T | T |
| FO-2.2 | 1/150 | T | T | T | T | C | A | T | C | A | T | T | T | A | C | A | T | T | T | C | A | G | T | C |
| FO-2.3 | 1/150 | T | T | C | T | C | A | C | C | A | C | T | T | A | T | A | T | T | T | C | A | G | T | T |
| FO-3.1 | 2/150 | T | T | T | T | C | A | T | C | A | T | T | T | A | T | A | T | T | T | T | A | G | T | T |
| FO-3.2 | 2/150 | T | T | T | T | C | A | T | C | G | T | T | T | A | T | A | T | T | T | C | A | G | T | T |
| FO-3.3 | 1/150 | T | C | T | T | C | A | T | C | A | T | T | T | A | T | A | C | T | T | C | A | G | T | T |
| FO-3.4 | 1/150 | T | T | T | T | C | A | T | C | A | T | A | T | A | T | G | T | T | T | C | A | A | T | T |
| FO-4.1 | 1/150 | T | T | T | C | C | A | T | C | A | T | T | C | A | T | A | T | T | T | C | A | G | T | T |
| FO* | 133/150 | T | T | T | T | C | A | T | C | A | T | T | T | A | T | A | T | T | T | C | A | G | T | T |

*Shared haplotype
